# Chromosomal aneuploidies induced upon Lamin B2 depletion are mislocalized in the interphase nucleus

**DOI:** 10.1007/s00412-016-0580-y

**Published:** 2016-02-27

**Authors:** Devika Ranade, Shivsmriti Koul, Joyce Thompson, Kumar Brajesh Prasad, Kundan Sengupta

**Affiliations:** Biology, Indian Institute of Science Education and Research, Pune, Main Building, Homi Bhabha Road, Pashan, Pune, Maharashtra 411008 India

**Keywords:** Aneuploidy, Chromosome territories, Lamins, Transcription, Cancer cells, Nucleus, Chromosome positioning

## Abstract

Chromosome territories assume non-random positions in the interphase nucleus with gene-rich chromosomes localized toward the nuclear interior and gene-poor chromosome territories toward the nuclear periphery. Lamins are intermediate filament proteins of the inner nuclear membrane required for the maintenance of nuclear structure and function. Here, we show using whole-genome expression profiling that Lamin A/C or Lamin B2 depletion in an otherwise diploid colorectal cancer cell line (DLD1) deregulates transcript levels from specific chromosomes. Further, three-dimensional fluorescence in situ hybridization (3D-FISH) analyses of a subset of these transcriptionally deregulated chromosome territories revealed that the diploid chromosome territories in Lamin-depleted cells largely maintain conserved positions in the interphase nucleus in a gene-density-dependent manner. In addition, chromosomal aneuploidies were induced in ~25 % of Lamin A/C or Lamin B2-depleted cells. Sub-populations of these aneuploid cells consistently showed a mislocalization of the gene-rich aneuploid chromosome 19 territory toward the nuclear periphery, while gene-poor aneuploid chromosome 18 territory was mislocalized toward the nuclear interior predominantly upon Lamin B2 than Lamin A/C depletion. In addition, a candidate gene locus *ZNF570* (Chr.19q13.12) significantly overexpressed upon Lamin B2 depletion was remarkably repositioned away from the nuclear lamina. Taken together, our studies strongly implicate an overarching role for Lamin B2 in the maintenance of nuclear architecture since loss of Lamin B2 relieves the spatial positional constraints required for maintaining conserved localization of aneuploid chromosome territories in the interphase nucleus.

**Electronic supplementary material:**

The online version of this article (doi:10.1007/s00412-016-0580-y) contains supplementary material, which is available to authorized users.

## Introduction

Chromosome territories (CTs) assume a unique sub-volume in the three-dimensional interphase nucleus. Gene-rich CTs are positioned toward the nuclear interior, while gene-poor chromosomes are closer to the nuclear periphery (Cremer et al. 2001; Croft et al. 1999). Human CTs 18 (~80 Mbp) and 19 (~59 Mbp) are of comparable DNA content, but of divergent gene densities of ~8.2 genes/Mbp and ~37.0 genes/Mbp, respectively. Chr.18 is peripheral, while Chr.19 is located toward the nuclear center in the interphase nucleus (Cremer et al. 2001; Croft et al. 1999). Such an arrangement is conserved in evolution, suggesting a functional significance for non-random chromosome positions (Neusser et al. 2007; Tanabe et al. 2002). Chromosome positioning is altered in biological processes such as senescence, adipocyte differentiation, spermatogenesis, immune responses, and DNA damage (Foster et al. 2005; Galiova et al. 2004; Mehta et al. 2007, 2013; Szczerbal et al. 2009).

The organization of CTs has been corroborated by high-throughput genomic approaches that map the physical proximity of chromatin contacts as a function of their ligation frequencies (van Berkum et al. 2010). Chromosome conformation capture studies (3C, 4C, 5C, and Hi-C) revealed enhanced *cis* interactions of sub-genomic regions of a chromosome in the nucleus, as opposed to interactions in *trans*, thereby also suggesting a territorial confinement of chromosomes (Kalhor et al. 2012). Moreover, computational contour maps from chromatin contact frequencies recapitulate a greater enrichment of gene-rich chromosomes toward the nuclear center (Kalhor et al. 2012; Lieberman-Aiden et al. 2009).

Gene loci are significantly smaller (~10^4^–10^6^-fold) than CTs and exhibit a non-random organization, since overexpressed genes typically “loop-out” of their CT (Chambeyron and Bickmore 2004; Volpi et al. 2000). Hi-C data revealed that the genome is organized in ~1 Mb sized topologically associated domains (TADs) that showed a higher cross-linking frequency with each other as compared to other regions of the genome (Dixon et al. 2012). Gene loci within a TAD have increased proximity in the human X chromosome as shown by three-dimensional fluorescence in situ hybridization (3D-FISH) (Nora et al. 2012), which suggested a concordance between single-cell imaging and 5C. However, the 3D localization of the Hox gene cluster as revealed by 3D-FISH showed a greater decompaction as compared to 5C approaches, suggesting a disagreement between single-cell versus population assays and reiterating the fundamental importance of microscopy-based single-cell assays in a context-specific manner (Williamson et al. 2014).

The molecular mechanisms that regulate chromosome positioning and genome organization in the interphase nucleus are largely unclear. Lamins, Lamin B receptor (LBR), emerin, actin, and CCCTC-binding Factor (CTCF) have been implicated in the maintenance of chromosome organization and chromatin contacts (Malhas et al. 2007; Meaburn et al. 2007; Ondrej et al. 2008; Phillips-Cremins et al. 2013; Shimi et al. 2008; Solovei et al. 2013; Taimen et al. 2009). Lamins serve to maintain nuclear structure and genome organization. Lamins are type V intermediate filaments that form coiled coil structures beneath the inner nuclear membrane (Goldman et al. 1986). In higher eukaryotes, A-type Lamins are encoded by a single gene *LMNA* (that codes for two splice variants—Lamin A and Lamin C) and B type (Lamin B1 and B2 are encoded by two different genes—*LMNB1* and *LMNB2*, respectively) (Hoger et al. 1990; Zewe et al. 1991). B-type Lamins are expressed during all stages of development, while A-type Lamins are expressed primarily in differentiated cells (Constantinescu et al. 2006; Rober et al. 1989). Laminopathies are a group of diseases due to mutations in Lamins, which show altered nuclear shapes, gene expression, and chromatin organization (Burke and Stewart 2002; Taimen et al. 2009). Both A- and B-type Lamins are involved in wide range of cellular processes such as replication, transcription, cell division, DNA damage repair, differentiation, and senescence (Butin-Israeli et al. 2015; Martin et al. 2010; Shimi et al. 2011; Shumaker et al. 2008; Spann et al. 2002; Swift et al. 2013; Tang et al. 2008). Furthermore, Lamins exhibit cell-type-specific expression levels and function (de Las Heras et al. 2013; Swift et al. 2013; Yang et al. 2011). In addition, the relative stoichiometry of A- and B-type Lamins is often cell-type specific and the ratio of A/B-type Lamins determines mechanical properties of nuclei (Swift et al. 2013).

Nuclear Lamins contribute to the organization of CTs by associating with chromatin in ‘Lamina-associated domains’ (LADs) during interphase (Guelen et al. 2008). LADs are enriched in Lamina-associated sequences (LASs) and are ~0.1–10-Mb regions characterized by low density of coding genes, high density of repetitive sequences, and inactive histone mark H3K9me2/3 (Belmont et al. 1993; Guelen et al. 2008; Harr et al. 2015; Towbin et al. 2012; Zullo et al. 2012). Constitutive LADs are conserved across cell types and correlate with a repressive state of chromatin (Meuleman et al. 2013). Live imaging assays performed on LADs using a fluorescently coupled m6A-Tracer reveal that they are dynamic, stochastic, and not solely confined to the nuclear periphery, suggesting roles for Lamins in the nuclear interior and periphery (Kind et al. 2013). CTs are mislocalized in cells expressing mutant Lamin A in progeria and cardiomyopathies (Meaburn et al. 2007; Mewborn et al. 2010). Lamin A and B1 depletion in fibroblasts and HeLa cells also showed mislocalized CTs (Malhas et al. 2007; Meaburn et al. 2007; Shimi et al. 2008; Tang et al. 2008).

Furthermore, Lamin A forms a complex with LAP2α and BAF and is involved in proper spindle orientation and assembly (Qi et al. 2015). B-type Lamins are also a part of the mitotic spindle matrix in *Xenopus laevis* and human cells (Goodman et al. 2010; Tsai et al. 2006). Lamin B2 maintains genomic stability and chromosome segregation in colorectal cancer cells (Kuga et al. 2014). Thus, Lamins are unique, since they are required for genome organization, chromosomal stability, and ploidy in mitosis. However, a rather unappreciated role for Lamins is in the spatial organization of diploid and aneuploid chromosome territories in the interphase nucleus.

Aneuploidy is a hallmark of several cancer and developmental disorders. In general, chromosomes assume a gene-density-based positioning pattern in cancer cells (Cremer et al. 2003). More specifically, cancer cells from several epithelial cancers are characterized by aneuploidy with a complex pattern of chromosomal gains and losses (Cimini and Degrassi 2005) that may show changes in chromosome positioning. Notably, CT18 and CT19 are more proximal to one another in colon and cervical cancer cell nuclei as compared to normal cells (Cremer et al. 2003). Chromosomal trisomies generated by artificial introduction of either gene-poor (Chr.7, Chr.18, peripheral) or gene-rich (Chr.19, central) chromosomes assume conserved locations in the nucleus consistent with their gene densities (Sengupta et al. 2007). Human X chromosome is altered from a predominantly central to a more peripheral location in X chromosome aneuploidies (XXXXY) (Petrova et al. 2007). Interestingly, while two copies of chromosome 21 territory are in closer proximity as compared to the third copy in cells derived from Down’s syndrome patients (Paz et al. 2013), spontaneous trisomy for Chr.12 in human embryonic stem cell line (WA09) also shows an altered position of the trisomic chromosome (Shete et al. 2014). However, given the overarching function of Lamins in regulating ploidy and genome organization, the specific role of Lamins in the spatial organization of aneuploid CTs is largely unclear.

Here, we have studied the role of Lamins in regulation of transcription and spatial organization of the genome in diploid DLD1 cells. Lamin B2 depletion in DLD1 cells shows chromosomal instability (CIN) (Kuga et al. 2014). We show that specific chromosomes are transcriptionally deregulated upon Lamin A/C and Lamin B2 knockdown. Remarkably, transcriptionally deregulated gene-rich or gene-poor chromosomes in Lamin-depleted diploid cells largely assume conserved chromosome positions as revealed by 3D-FISH. However, aneuploid chromosomes were mislocalized in sub-populations of Lamin B2 and not Lamin A/C-depleted cells. In addition, candidate gene loci were repositioned upon Lamin B2 depletion, consistent with an increase in their gene expression levels. Taken together, we propose the involvement of Lamin B2 in mechanisms that regulate spatial organization of aneuploid CTs in the interphase nucleus.

## Materials and methods

### Cell culture

DLD1 colorectal adenocarcinoma cells were obtained from the lab of Thomas Ried, NCI/NIH, Bethesda, USA, and karyotyped independently by 4′,6-diamidino-2-phenylindole (DAPI) to ascertain karyotypic stability across passages. DLD1 cells were grown in RPMI 1640 media (Invitrogen, Cat. No. 11875) supplemented with 10 % fetal bovine serum (FBS, Invitrogen, Cat. No. 6140-079 Carlsbad, USA) and antibiotics penicillin (100 U/mL) and streptomycin (100 μg/mL) (Invitrogen, Cat. No. 15070-063) at 37 °C with 5 % CO_2_.

### Small interfering RNA transfection

The sequences of the small interfering RNA (siRNA) oligonucleotides targeting Lamins are as follows: *LMNA/C*: 5′-CAGUCUGCUGAGAGGAACA-3′, *LMNB2*: 5′- GAGCAGGAGAUGACGGAGA-3′, *LMNB1*: 5′- AGACAAAGAGAGAGAGAUG-3′ and *LMNB2* scrambled: 5′-GGAAGCGUAGACGGAAGAG-3′. DLD1 cells were transfected with 100 nM siRNA oligonucleotide using Lipofectamine RNAiMax (Invitrogen 13778-075) in media with reduced serum (OptiMEM, Invitrogen Cat. No. 31985-070). Control siRNAs used were On-target Plus non-targeting siRNA controls (Dharmacon-D-001810-01-20, D-001810-02-20) or siLacZ: 5′-CGUACGCGGAAUACUUCGA-3′; positive control is a siRNA against the gene *PLK1* (si*PLK1*)—polo-like kinase—5′-UGACCUACAUCGACGAGAA-3′. The uptake of transfection mix was for 6 h, followed by replenishment of complete growth medium. The total duration of the knockdown was for 48 h at 37 °C.

### Western blotting

Cell lysates were prepared using radioimmunoprecipitation assay (RIPA) buffer and quantified using bicinchoninic acid (BCA) kit (Pierce, Cat. No. 23225). Equal amounts of the protein were boiled in 4× Laemmli buffer and resolved on a 10 % acrylamide–bisacrylamide gel. The protein was transferred to an activated PVDF membrane at a constant voltage of 90 V for 90 min. The membrane was blocked in 5 % non-fat dried milk prepared in 1× Tris-buffered saline–Tween 20 (1× TBST) for 1 h at room temperature (RT). Primary antibodies used were as follows: Rabbit anti-Lamin A/C (Epitomics (2966-S), 1:5000), Rabbit anti-Lamin B1 (Abcam (ab16048), 1:1000), Mouse anti-Lamin B2 (Abcam (ab8983), 1:400), Mouse anti-Actin (Abcam (ab3280), 1:400), and Rabbit anti-GAPDH (Sigma (G9545), 1:5000). Antibody dilutions were prepared in 0.5 % non-fat dried milk in 1× TBST and incubated overnight at 4 °C or for 3 h at RT. Secondary antibodies used were sheep anti-mouse IgG–horseradish peroxidase (HRP) (GE cat no NA9310V, 1:5000) and donkey anti-rabbit IgG HRP (GE NA9340V, 1:10,000) for 1 h at RT. Blots were developed using chemiluminescent substrate GE ECL Prime (89168-782), Thermo ECL Western Blot Substrate (32132), and images were acquired at incremental exposures of 10 s under a chemiluminescence system (LAS4000, GE).

### Immunofluorescence assay

Cells grown on coverslips (18 × 18 or 22 × 22 mm^2^) were briefly washed using 1× phosphate-buffered saline (PBS, pH 7.4) (5 min, twice at RT) followed by cold cytoskeletal (CSK) buffer (0.1 M NaCl, 0.3 M sucrose, 3 mM MgCl_2_, 10 mM PIPES (pH 7.4), 0.5 % Triton X-100) treatment on ice for 5 min. Cells were fixed in 4 % paraformaldehyde (PFA) (prepared in 1× PBS, pH 7.4) for 10 minutes at RT followed by permeabilization in 0.5 % Triton X-100 (prepared in 1× PBS) for 10 min. Blocking was performed for 30 min using 1 % bovine serum albumin (BSA) in 1× PBS at RT. Primary antibodies used were as follows: Rabbit anti-Lamin A/C (Epitomics (2966-S), 1:300), Rabbit anti-Lamin A (Abcam (ab26300), 1:500), Rabbit anti-Lamin B1 (Abcam (ab16048), 1:500), Mouse anti-Lamin B2 (Abcam (ab8983), 1:600), and Mouse anti-Lamin A (Abcam (ab8980), 1:500). Antibody dilutions were prepared in 0.5 % BSA in 1× PBS and incubated for 90 min at RT. Secondary antibodies used were goat anti-rabbit IgG–Alexa 488 (Invitrogen (A-11034) 1:1000) and goat anti-mouse IgG–Alexa 568 (Invitrogen (A-11004) 1:1000) for 1 h at RT. Cells were counter stained with DAPI for 2 min at RT, washed in 1× PBS, mounted in Slowfade Gold Antifade (Invitrogen S36937), and stored in 4 °C until they were imaged. Quantification of fluorescence intensities of the acquired images was performed using ImageJ with line scans manually drawn across nuclei.

### Three-dimensional fluorescence in situ hybridization—chromosome territories

#### Fixation and permeabilization

Cells were grown to a confluency of ~30–40 % on glass coverslips (18 × 18 or 22 × 22 mm^2^) placed in single wells of a six-well plate and were subjected to siRNA knockdown (Kd). Cells were washed three times in 1× PBS for 5 min each at RT. The cells were incubated on ice for 5 min in pre-chilled CSK buffer and immediately fixed in 4 % paraformaldehyde (PFA, prepared in 1× PBS (pH 7.4)) for 7 min at RT. The cells were washed in 0.1 M Tris-HCl (pH 7.4) followed by two washes with 1× PBS for 5 min each at RT. The cells were repermeabilized in 0.5 % Triton X-100 (prepared in 1× PBS) for 10 min and incubated in 20 % glycerol (prepared in 1× PBS) for 60 min followed by four freeze–thaw cycles in liquid nitrogen. The cells were washed three times in 1× PBS for 5 min each and incubated in 0.1 N HCl for 10 min followed by three washes in 1× PBS for 5 min each. The cells were stored in 50 % formamide (FA)/2× saline sodium citrate (SSC) (pH 7.4) overnight at 4 °C or until used for hybridization.

#### Hybridization

Chromosome painting probes were obtained from Applied Spectral Imaging (ASI), Israel, or MetaSystems, USA. Probes were equilibrated at 37 °C for 5 min followed by denaturation at 80 °C for 5 min and quick chilled on ice for 2 min followed by a pre-annealing at 37 °C for 30 min. This denatured probe (3–4 μL) was spotted onto fixed cells, sealed, and subjected to co-denaturation at 80 °C for 5 min. Hybridization was for 48 h in a humidified box at 37 °C.

#### Detection

Post hybridization, coverslips were washed in 50 % FA/2× SSC (pH 7.4), thrice for 5 min each at 45 °C, followed by three washes for 5 min each in 0.1× SSC at 60 °C with gentle agitation. Coverslips were briefly rinsed in 0.1 % Tween 20/4× SSC and counterstained with DAPI for 2 min, washed in 2× SSC and mounted in Slowfade Gold Antifade (Invitrogen S36937), and stored at 4 °C until they were imaged.

#### Imaging

Image acquisition was performed on a Zeiss LSM 710 or 780 confocal microscope (Carl Zeiss, Thornwood, NJ, USA) with a ×63 Plan-Apochromat 1.4 NA oil immersion objective using scan zoom of 2.5. Acquisition of Z-stacked images (voxel size of 0.105 μm × 0.105 μm × 0.34 μm) was at 512 × 512 pixels per frame using 8-bit pixel depth for each channel. The line averaging was set to 4, and images were collected sequentially in a three-channel mode.

### Three-dimensional immunofluorescence in situ hybridization-gene loci

#### Preparation of bacterial artificial chromosome DNA probes

Bacterial artificial chromosome (BAC) DNA was extracted from clones purchased from CHORI BACPAC Resources (using Hi-Pure Plasmid DNA Extraction Kit (Invitrogen K210017). BAC clones used were RP11-1134K12 for *ZNF570* gene. This DNA was nick translated using fluorescently labeled dUTP (Abbott) and Nick Translation Mix (Roche 11 745 808 910). Labeling reaction was at 15 °C for 90 min, followed by termination of reaction using 0.5 M EDTA (pH 8.0) at 65 °C for 10 min. The DNA was precipitated using ethanol and 3 M sodium acetate and resuspended using deionized FA and Master Mix containing dextran sulfate and 2× saline sodium citrate (SSC) buffer.

#### Fixation and permeabilization of cells

Cells grown on coverslips and siRNA treated for 48 h were washed twice (5 min each) with 1× PBS (pH 7.4) followed by permeabilization on ice using ice-cold CSK buffer containing 0.5 % Triton X-100 for 5 min. Cells were subsequently fixed in 4 % paraformaldehyde (PFA) solution prepared in 1× PBS for 5–7 min at RT. The fixation protocol followed was the same as that for 3D-FISH (described above in 3D-FISH for visualizing CTs).

#### Immunostaining followed by hybridization

Previously fixed coverslips were subjected to two washes with 1× PBS (5 min each). Blocking was performed for 30 min using 1 % bovine serum albumin (BSA) in 1× PBS at RT. Primary antibodies used were as follows: Mouse anti-Lamin B2 (Abcam (ab8983), 1:600) and Rabbit anti-Lamin A (Abcam (ab26300), 1:500). Antibody dilutions were prepared in 0.5 % BSA in 1× PBS and incubated for 90 min at RT. Secondary antibodies used were goat anti-mouse IgG–Alexa 633 (Invitrogen (A-21052) 1:1000) and goat anti-rabbit IgG–Alexa 488 (Invitrogen (A-11034) 1:1000) for 1 h at RT. Post fixation and post permeabilization were subsequently performed with 4 % PFA (5 min RT) and 0.5 % Triton X-100 in 1× PBS (5 min RT), respectively. This was followed by two washes each with 1× PBS (5 min each) and 50 % FA/2× SSC (pH 7.4). The probe for *ZNF570* was equilibrated at 37 °C for 5 min followed by denaturation at 80 °C for 5 min and quick chilled on ice for 2 min. Pre-annealing was performed at 37 °C for 30–40 min. This denatured probe (3–4 μL) was spotted onto the fixed cells that were subjected to immunostaining for Lamin A and Lamin B2 and subjected to co-denaturation at 80 °C for 5 min. Hybridization was for 48 h in a humidified box at 37 °C.

#### Post hybridization washes

Post hybridization, coverslips were washed in 50 % FA/2× SSC (pH 7.4), thrice for 5 min each at 45 °C, followed by three washes for 5 min each in 0.1× SSC at 60 °C with gentle agitation. Coverslips were stained with DAPI and mounted with Antifade and stored in 4 °C until they were imaged. The images were acquired on Zeiss LSM 710 or 780 confocal microscope similarly as for CT hybridizations, using a zoom = 2.0.

### Radial distance measurements of chromosome territories

Three-dimensional distance measurements of CTs were performed using Image-Pro Plus (v 7.1), Media Cybernetics, USA. Briefly, LSM files containing optical sections (*z* = 0.34 μm) of the hybridized nuclei were subjected to 3D surface rendering. Three-dimensional reconstructions of each nucleus were performed on individually cropped nuclei. The acquired images were thresholded and surface rendered for each of the red, green, and blue channels. The geometric center of the DAPI-stained nucleus (blue channel) and the CTs (red and green channels) were determined using plugins from the software, and the distance between the geometric center of the nucleus (A) and that of the territory (B) was measured (X). The vector from the geometric center of nucleus (A) to the geometric center of the CT (B) was extended to a third collinear point at the nuclear periphery (C). The distance between the geometric center (A) of the nucleus and (C) was calculated (Y). The relative distance of a CT from the center of the nucleus was calculated as a percentage of the total distance from the center of the nucleus to the nuclear periphery (Y), %radial distance (RD) = (*X*/*Y*) ∗ 100 (Tanabe et al. 2002).

### Measurements for spatial organization of gene loci

#### Distance measurement from the nuclear periphery

Distances of gene loci from the nuclear periphery were measured (in μm) in 3D using Lamin A signal at the nuclear periphery to demarcate the edge of the nucleus. Briefly, surface rendering was performed for the gene locus (red channel), Lamin A (green channel), Lamin B2 (far-red channel), and the nucleus (DAPI signal blue channel) using Huygens Professional software. For Lamin B2 knockdown (Kd) cells, the nuclei which showed a depletion of Lamin B2 (far-red channel) were used for analysis. Briefly, surface rendering for Lamin A was used as an anchor for measurements. Center of mass (CM) was determined for the gene locus signal of interest, and the closest distance between the CM and surface of the anchor (Lamin A signal) was measured.

### Statistical analysis

Graphs were plotted using Graph Pad Prism 5.0 and Sigma Plot 12.0. Statistical comparisons were performed using Graph Pad Prism 5.0 software. Fisher’s exact and χ^2 ^ test was used to compare the distribution of % radial distances (RD) of CTs binned in five nuclear sub-shells. For comparison of volumes of CTs and the nucleus between diploid and aneuploid sub-populations, non-parametric ANOVA Kruskal–Wallis test was used. The distance of gene locus *ZNF570* from the lamina was compared using two-sample Kolmogorov–Smirnov (KS) test between control and Lamin B2-depleted cells; *p* value <0.05 was considered to be statistically significant.

### RNA extraction and quantitative reverse transcription-PCR

RNA extraction was performed using PureLink RNA Mini Kit (12183018A). cDNA was synthesized using ImProm II Reverse Transcriptase system (Promega A3800). Quantitative real-time PCR was performed using SYBR Green (SAF Labs). Sequences of primers are in Table S1. *ACTIN* and *GAPDH* served as internal controls.

### Gene expression profiling using microarrays

Briefly, RNA was extracted from three independent biological replicates each from control (untreated), Lamin A/C Kd, and Lamin B2 Kd DLD1 cells, and its quality was ascertained using bioanalyzer (Agilent). Cy3-labeled complementary RNA was prepared using T7 promoter-based linear amplification (Agilent Quick Amp Labeling Kit, Cat No. 5190–0442). Qiagen RNeasy Mini Kit (Cat No. 74104) was used for RNA purification. Hybridization was performed using a human 8× 60K array (Agilent single-color 27114) and labeled using Agilent’s in situ hybridization kit (Cat No. 5188-5242). Array scanning was performed, and data acquired was normalized (50th percentile shift normalization). Analyses of gene expression levels were based on comparison of hybridizations between knockdown and control (untreated DLD1 cells) as reference. Gene expression levels (absolute fold change ≥2.0, *p* < 0.05) were used for further analyses. The microarray data can be accessed using GEO Accession number GSE73269. The % deregulation per chromosome was calculated as (no. of deregulated genes on a chromosome / total no. of coding genes on that chromosome) × 100. Number of coding genes and size of chromosomes were obtained from MapViewer (NCBI). Chromosomes showing >0.7 % deregulation were considered significant since this represents >50 % of the total extent of transcriptional deregulation.

### Preparation of metaphase spreads

Cells (control, Lamin Kd) were blocked in metaphase using Colcemid (0.1 μg/mL) for 90 min. Hypotonic treatment (0.075 M KCl) was performed for 30 min at RT, followed by fixation in five to six drops of fixative (methanol/acetic acid 3:1), followed by three washes in fixative. Cells were dropped onto glass slides, and metaphases were stained with DAPI, imaged, and counted.

### Fluorescence-activated cell sorting analysis by propidium iodide staining

Cells (control, Lamin Kd) were fixed in 70 % ethanol (in 1× PBS), subjected to RNase treatment along with propidium iodide staining (1 h) on ice. Cell suspensions were subsequently run on FACSCalibur (BD Biosciences).

### RNA fluorescence in situ hybridization

#### Probe preparation

The probe for RNA FISH for *ZNF570* was prepared by nick translation of the BAC clone (“Preparation of bacterial artificial chromosome DNA probes” section–3D FISH for gene loci above). Upon precipitation, the DNA was resuspended in 8 μL of deionized FA and stored at −20 °C till further use. For hybridization, the probe was equilibrated at 37 °C for 5 min followed by denaturation at 80 °C for 5 min, mixed with equal volume of 2× hybridization mix containing vanadyl ribonucleoside complex, and incubated on ice for 30 min.

#### Fixation and hybridization

Cells were washed twice in 1× PBS (5 min each), followed by treatment with CSK buffer on ice for 5 min and fixation using 4 % PFA (7 min RT). The cells were washed with 70 % ethanol twice and stored at −20 °C until further use. Prior to hybridization, the cells were subjected to an ethanol series (70–90–100 % ethanol) and air-dried. The probe was added to cells and incubated at 37 °C overnight, followed by washes with 50 % FA/2× SSC and 2× SSC (pH 7.2-7.4) at 42 °C (three washes each of 5 min each). The cells were mounted using DAPI–Antifade. All reagents for RNA FISH were prepared using DEPC-treated water (Chaumeil et al. 2008).

## Results

### Specific chromosomes are transcriptionally deregulated upon Lamin A/C and Lamin B2 depletion

Lamins are filamentous proteins that localize beneath the inner nuclear membrane and maintain nuclear structure and function (Adam and Goldman 2012). In addition to the nuclear periphery, Lamins exist in the nucleoplasm as a meshwork that extends into the nuclear interior and associate directly or indirectly with LAP2α, BANF1, PCNA, and transcription factors (Dechat et al. 2000; Lee et al. 2001; Shumaker et al. 2008). The role of Lamins is therefore not just confined to the nuclear periphery but significantly extends to the nuclear interior, owing to its interaction with proteins at both these nuclear locations (Gruenbaum and Medalia 2015; Kolb et al. 2011). A- and B-type Lamins are implicated in DNA replication and repair, transcription, and senescence (Moir et al. 1994; Shimi et al. 2011; Shumaker et al. 2008; Tang et al. 2008).

**Fig. 1:**
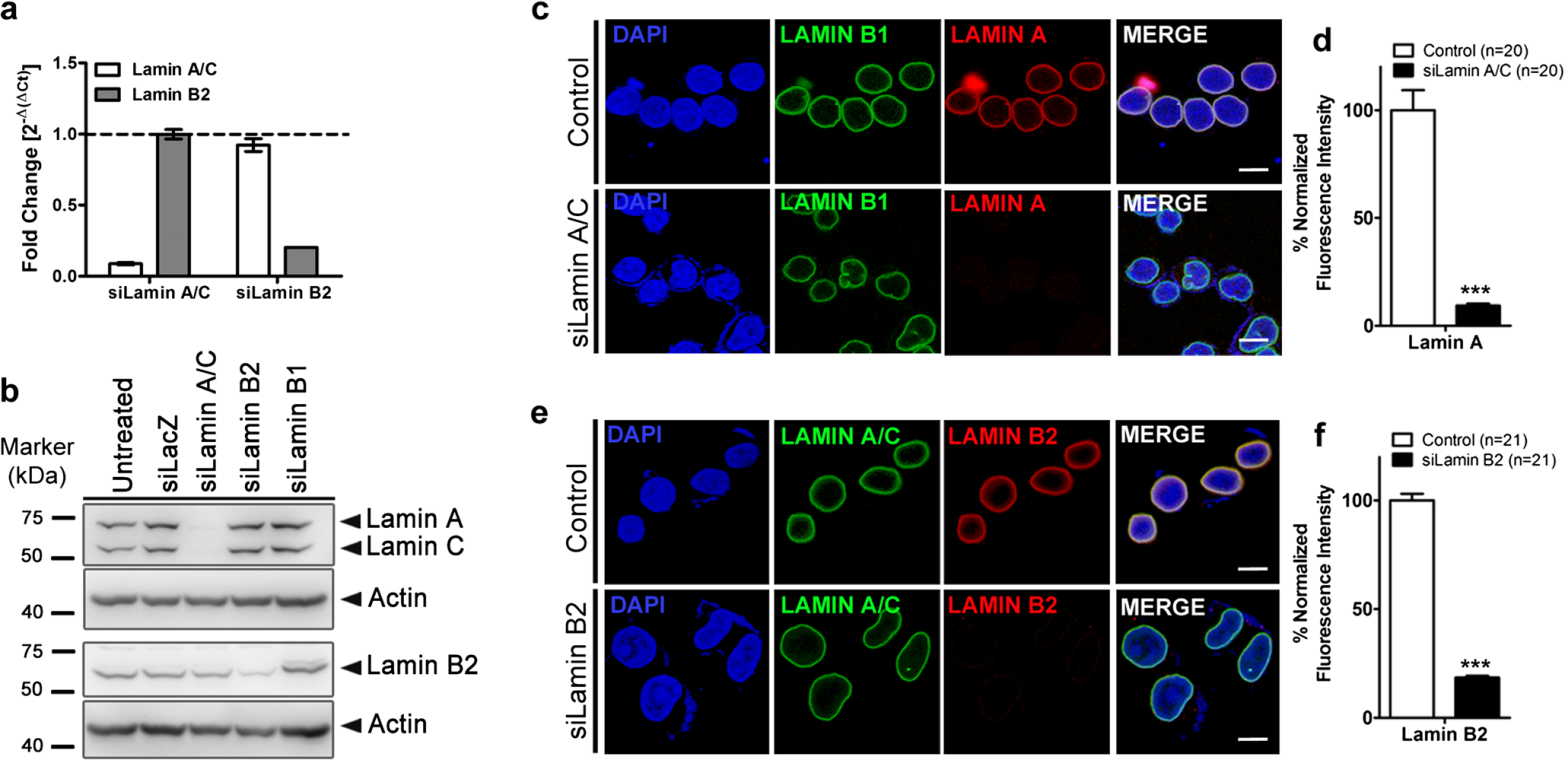
Depletion of Lamin A/C and B2 in DLD1 cells. **a** qRT-PCR showing siRNA-mediated knockdown of Lamin A/C and Lamin B2 in DLD1 cells, normalized to expression levels of *ACTIN* and compared to non-targeting control (siLacZ). Data shown is a representative out of three independent biological replicates. *Error bars* represent standard error of mean (SEM). **b** Western blots showing knockdowns of Lamin A/C and Lamin B2 in DLD1 cells. Depletion of Lamin A/C does not impact the expression of Lamin B2 and vice versa. Loading control: actin. Data shown is a representative result from five independent experiments. **c** Immunostaining of Lamin B1 (*green*) and Lamin A (*red*) in control (untreated) and Lamin A/C Kd cells. Representative out of four independent experiments. *Scale bar* ~10 μm. **d** Quantification of fluorescence intensity performed by line scans across nuclei from **c. e** Immunostaining of Lamin A/C (*green*) and Lamin B2 (*red*) in control (untreated) and Lamin B2 Kd cells. *Scale bar* ~10 μm. **f** Quantification of fluorescence intensities performed by line scans across nuclei from **e**. *Error bars* in **d**, **f** represent SEM, *n* number of nuclei Knockdown of Lamin A and Lamin B2 was ~85–90 % at the single-cell level (*p* < 0.001, unpaired Student’s *t* test)

**Fig. 2:**
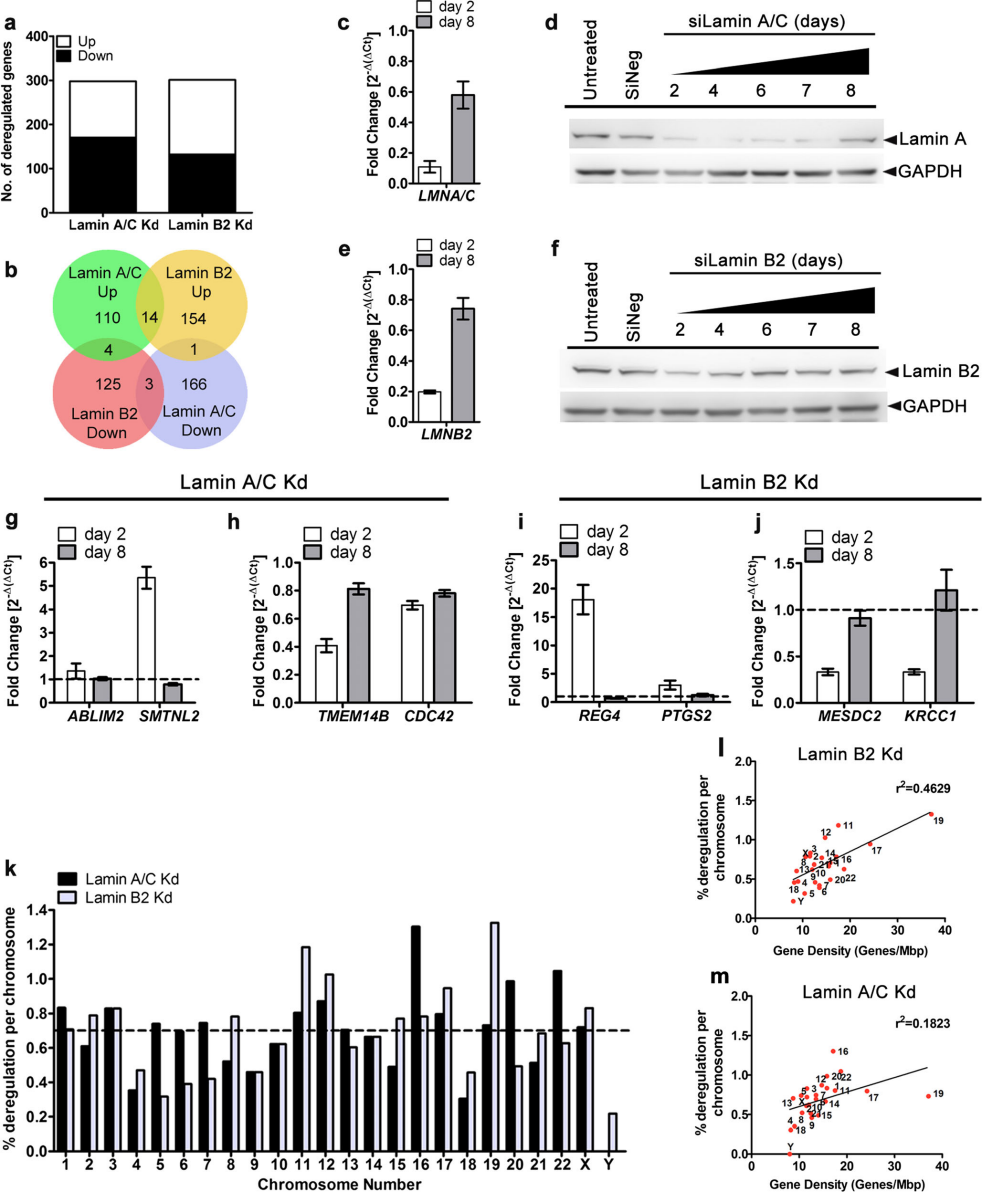
Impact of Lamin A/C and B2 depletion on the transcriptome. **a** Number of deregulated genes (up and down) upon Lamin A/C and Lamin B2 depletion (cutoff ≥2.0-fold on absolute scale) from genome-wide expression analyses. **b** Common and uniquely deregulated genes upon Lamin A/C and Lamin B2 depletion. Lamin A/C or B2 Kd reveals ~300 genes that were deregulated (Lamin A/C Kd-128 genes up, 170 genes down and Lamin B2 Kd-169 genes up, 132 genes down). **c** qRT-PCR showing recovery of Lamin A/C transcript at the end of 8 days, normalized to expression of *ACTIN*. **d** Western blot showing levels of Lamin A when DLD1 cells treated with siLamin A/C were grown for 2–8 days to assess recovery from siRNA-mediated knockdown. Loading control: GAPDH. **e** qRT-PCR showing recovery of Lamin B2 transcript at the end of 8 days, normalized to expression of *ACTIN*. **f** Western blot showing levels of Lamin B2 when DLD1 cells treated with siLamin B2 were grown for 2–8 days to assess recovery from siRNA-mediated knockdown. Loading control: GAPDH. Data shown in **d**, **f** are representative results from two independent experiments. **g** Expression levels of genes upregulated upon Lamin A/C Kd (*ABLIM2*, *SMTNL2*) at the end of day 2 were restored at day 8. **h** Genes downregulated upon Lamin A/C Kd (*TMEM14B*, *CDC42*) at the end of day 2 recovered at day 8. **i** Expression levels of genes upregulated upon Lamin B2 Kd (*REG4*, *PTGS2*) at the end of day 2 were restored at day 8. **j** Genes downregulated upon Lamin B2 Kd (*MESDC2*, *KRCC1*) at the end of day 2 recovered at day 8. Data for all qRT-PCRs (**c**, **e**, **g**–**j**) is a compilation of two biological replicates and normalized to expression levels of *ACTIN. Error bars* represent SEM. **k** Genes deregulated per chromosome upon depletion of Lamin A/C and Lamin B2 in DLD1 cells. The % deregulation = (no. of deregulated genes on a chromosome / total no. of coding genes on that chromosome) × 100. Details of number of coding genes and size of chromosomes were obtained from MapViewer (NCBI). **l**, **m** Lamin B2 Kd (*r* ^2^ = 0.4629) shows greater correlation between transcriptional deregulation and gene density as compared to Lamin A/C Kd (*r* ^2^ = 0.1823)

We sought to examine the impact of Lamin depletion on the transcriptome and spatial organization of chromosomes in otherwise diploid colorectal cancer cells (DLD1), which are karyotypically stable across passages, distinguishing the same from other cell lines with severe chromosomal aberrations. We first established the conditions for Lamin depletion in DLD1 cells by performing siRNA-mediated knockdowns (Kd). Lamin Kd showed a viability of ~60–70 % in DLD1cells (Fig. S1a). A knockdown of >80–90 % was ascertained at the transcript level by quantitative reverse transcription (qRT)-PCR (Fig. 1a), protein level by immunoblotting (Figs. 1b and S1b–d), and in single cells by immunofluorescence staining (Fig. 1c–f). Immunoblots revealed that Lamin A/C Kd did not significantly affect the levels of B-type Lamins (Figs. 1b and S1b). Likewise, depletion of Lamin B1 or B2 did not affect Lamin A/C levels (Fig. 1b). The exclusivity of the knockdowns enabled us to interpret the consequences of a single Lamin depletion. This is noteworthy since expression levels and stoichiometry of Lamins are important determinants of Lamin localization and function in a cell-type-dependent manner (Guo et al. 2014; Shimi et al. 2015; Swift et al. 2013). Since the extent of siRNA-mediated Lamin B1 depletion (~50-60 %) was not as much as Lamin A/C and B2 in our hands, we focused on examining the effects of Lamin A/C and Lamin B2 knockdowns (Figs. 1b and S1b). Immunostaining showed ~85–90 % depletion of Lamin A and B2 at the single-cell level (Fig. 1d, f). Counterstaining with Lamin B1 in Lamin A/C-depleted cells and Lamin A/C upon Lamin B2 Kd revealed distorted nuclear shapes, nuclear invaginations, blebbing, and nuclear furrows (Fig. S2) as found in Lamin A/C- and B1-depleted HeLa cells and in cells derived from laminopathies (Shimi et al. 2008; Taimen et al. 2009). Independently, Lamin A/C and B2 Kd were performed in DLD1 cells, followed by whole-genome expression profiling using gene expression arrays in three independent biological replicates. The data analyses revealed ~300 genes that were significantly deregulated (inclusive of upregulation and downregulation) in either Lamin A/C- or Lamin B2-depleted cells (absolute fold change ≥2.0-fold) (Fig. 2a). Remarkably, in Lamin A/C Kd, 276 out of 298 genes were uniquely deregulated, while in Lamin B2 Kd, 279 out of 301 genes were uniquely deregulated (Fig. 2b). In both Lamin A/C- and B2-depleted cells, 22 genes (~7 %) were commonly deregulated (Fig. 2b). Gene expression levels from expression arrays were independently validated using qRT-PCR for the top deregulated genes (absolute fold change ~2–6-fold). This showed a fold change in the same direction as that obtained from the microarray data set (Fig. S3a, b). We next performed a recovery assay where DLD1 cells were allowed to recover from the Lamin knockdowns by growing cells up to day 8, well beyond the 48-h duration of the knockdown. At the end of day 8, there was ~60–70 % recovery in the transcript and protein levels of Lamin A/C and B2, respectively (Fig. 2c–f). We detected a significant recovery in the transcript levels of candidate genes *ABLIM2* and *SMTNL2* upregulated in Lamin A/C Kd and *TMEM14B* and *CDC42* downregulated in Lamin A/C Kd (Fig. 2g, h). Recovery was also detected for *REG4* and *PTGS2* upregulated in Lamin B2 Kd and *MESDC2* and *KRCC1* downregulated upon Lamin B2 Kd (Fig. 2i, j).

Whole-genome expression profiling using microarrays from Lamin A mutant (E161K) human cardiomyocytes showed deregulated gene expression levels predominantly from gene-poor chromosome 13 (Mewborn et al. 2010). Likewise, mouse fibroblasts lacking functional Lamin B1 revealed transcriptionally deregulated clusters on the peripherally positioned mouse chromosome 18 (Malhas et al. 2007). These evidences prompted us to investigate if specific chromosomes were transcriptionally deregulated upon Lamin A/C or B2 depletion. From our expression array data sets independently derived from Lamin A/C and Lamin B2 knockdowns, we calculated the extent of transcriptional deregulation for each chromosome by expressing the total number of deregulated genes on a chromosome as a fraction of the total number of coding genes from that chromosome (Fig. 2k). Chromosomes showing >0.7 % deregulation in transcript levels were shortlisted. Chromosomes showing significant deregulation upon Lamin A/C Kd were Chr.16, 22, 20, 12, 1, 3, 17, and 19 (descending order of expression deregulation). While upon Lamin B2 Kd, major deregulated chromosomes were Chr.19, 11, 12, 17, X, 8, 3, and 2 (descending order of expression deregulation) (Fig. 2k). Comparatively neither Lamin A/C Kd nor Lamin B2 Kd showed a significant transcriptional deregulation from gene-poor chromosomes 13, 18 or 7 (whole chromosomes), although these chromosomes are proximal to the nuclear lamina (Fig. 2k). A plot of % transcriptional deregulation against gene density showed a higher correlation with gene density upon Lamin B2 Kd (*r* ^2^ = 0.4629) as compared to Lamin A/C Kd (*r* ^2^ = 0.1823) (Fig. 2l, m). This reveals an enhanced transcriptional deregulation from gene-rich chromosomes upon Lamin B2 Kd. Taken together, gene expression profiling of Lamin A/C- or B2-depleted cells uncovered specific chromosomes that were significantly deregulated in their gene expression levels (Fig. 2k).

### Lamin depletion induces chromosomal aneuploidies

**Fig. 3:**
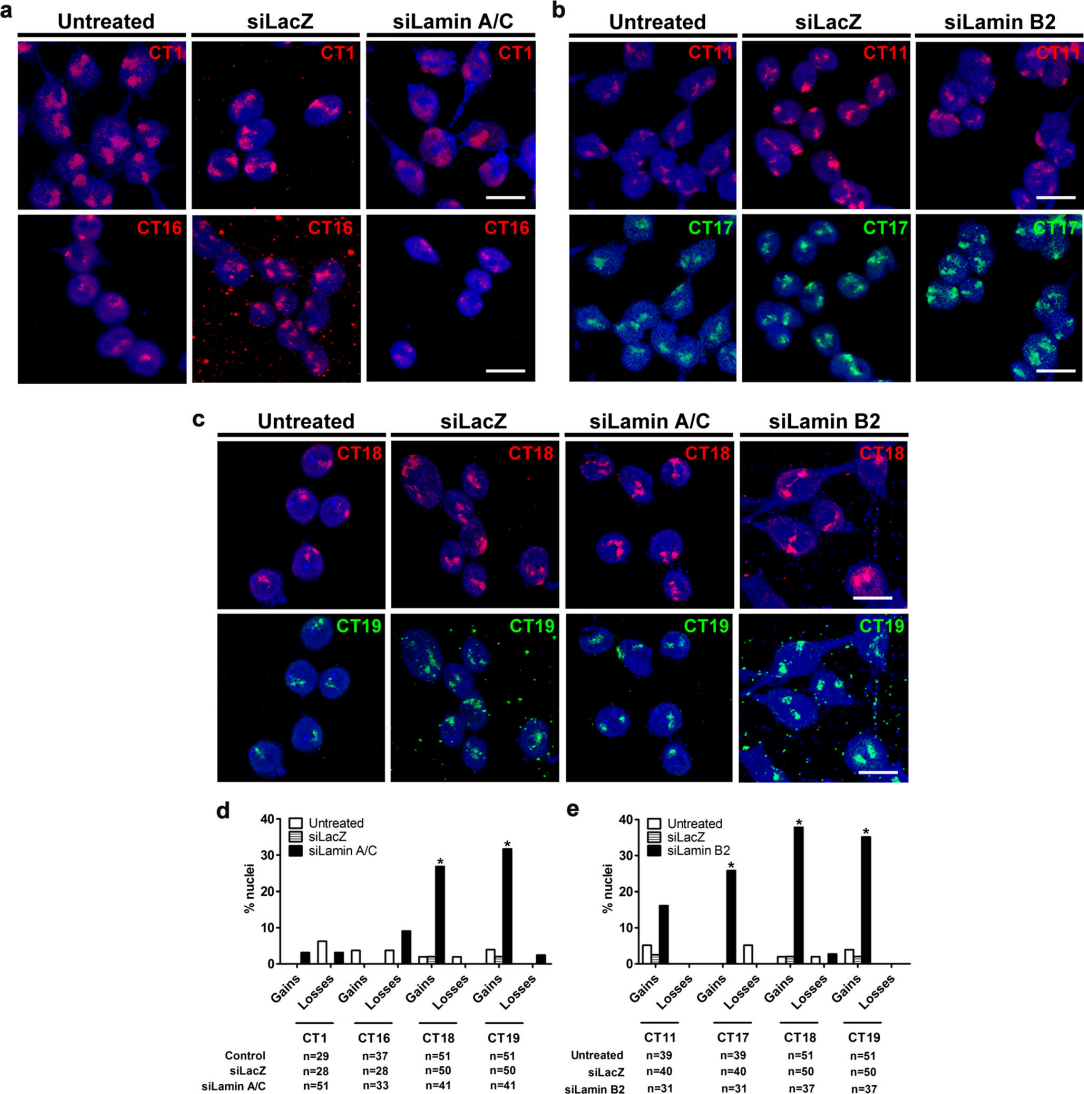
Lamin depletion induces chromosomal aneuploidies. **a** Merged maximum intensity projection of confocal image stacks of chromosome territories (CT)—CT1 and CT16 for control (untreated and siLacZ treated) and siLamin A/C-treated DLD1 cells. **b** Merged maximum intensity projection of confocal image stacks of chromosome territories (CT)—CT11 and CT17 for control (untreated and siLacZ treated) and siLamin B2-treated DLD1 cells. **c** Merged maximum intensity projection of confocal image stacks of chromosome territories (CT)—CT18 and CT19 for control (untreated and siLacZ treated), siLamin A/C-, and siLamin B2-treated DLD1 cells. Nuclei are stained with DAPI, hybridizations for CT1 and CT16 were single-color hybridizations, while CT11–CT17 and CT18–CT19 were dual color hybridizations. *Scale bar* ~10 μm. **d** Quantification of number of chromosome territories in interphase nuclei of control and Lamin A/C-depleted cells. Number of nuclei scored in each treatment (*n*) ~28–51. Quantification is a representative result out of two biological replicates. Chromosomes 18 and 19 are aneuploid (in ~20–25 % cells) upon Lamin A/C depletion. **e** Quantification of number of chromosome territories in interphase nuclei of control and Lamin B2-depleted cells. Chromosomes 11 and 17 (aneuploid in ~15–25 % cells) and chromosomes 18–19 (aneuploid in ~30–40 % cells) upon Lamin B2 depletion. Number of nuclei scored in each treatment (*n*) ~28–51. Quantification is a representative result out of two biological replicates

We next performed three-dimensional fluorescence in situ hybridization (3D-FISH) of specifically those chromosomes that showed greater transcriptional deregulation over others independently in Lamin A/C and B2 depletion. Chromosome territories (CTs) 1 and 16 were examined upon Lamin A/C Kd, and CTs 11 and 17 were examined upon Lamin B2 Kd (Fig. 3a, b). In addition, the most gene-rich chromosome 19 that was deregulated independently in Lamin A/C and B2 Kd was also examined along with gene-poor chromosome 18 of comparable DNA content (Fig. 3c). Furthermore, chromosome 18 showed a relatively lower (~0.5–0.6 %) transcriptional deregulation in either Lamin A/C or B2 Kd (Fig. 2k). Three-dimensional FISH showed that Chr.1, 11, 16, 17, 18, and 19 were diploid in ~95 % control cells (untreated cells and cells treated with non-targeting siRNA (siLacZ)) (Figs. 3d, e and S4a–i). However, while imaging Lamin Kd nuclei, we consistently detected sub-populations (~20–30 % of cells) with more than two CTs (Fig. 3d, e). Chromosomes 18 and 19 were gained (three to four copies) in Lamin A/C Kd cells (~20–30 %) (Figs. 3d and S4c, d), while chromosomes 11, 17, 18, and 19 were gained (three to four copies) in Lamin B2 knockdown cells (Figs. 3e and S4e–h). We also tested for the extent of aneuploidies of chromosomes that were not transcriptionally deregulated and therefore not shortlisted in each of Lamin A/C (CT11, 17) or B2 Kd (CT1, 16) cells. Chromosome 17 was gained (three copies) in Lamin A/C Kd in ~20 % cells, while chromosomes 1 and 16 were not gained upon Lamin B2 Kd (Fig. S4i). Immuno-FISH assays showed a comparable depletion of ~75–80 % of Lamin levels in both the diploid and aneuploid sub-populations of cells (Fig. S5). A scrambled (non-targeting) siRNA oligonucleotide control of Lamin B2 did not show aneuploidy for CT18 and 19, underscoring the specificity of the siRNA against Lamin B2 in DLD1 cells (Fig. S4j, k). In addition, an shRNA sequence against Lamin B2 showed a comparable extent of aneuploidy (>30 %) (data not shown), suggesting that Lamin-depletion-induced aneuploidies were simply not a manifestation of transient siRNA-mediated knockdowns.

We performed fluorescence-activated cell sorting (FACS) profiling to ascertain the ploidy of Lamin-depleted DLD1 cells (Fig. S6a–d). This profile showed a comparable proportion of cycling cells in the control and Lamin Kd populations. In addition, we did not detect polyploid sub-populations in control or Lamin Kd cells (Fig. S6a–d). Further, the levels of the major cell cycle checkpoint proteins Chk1 and Chk2 remained unaffected (Fig. S6e, f). To assess the effect of Lamin depletion on chromosomal instability (CIN), we examined metaphase spreads derived from Lamin knockdown cells (Fig. S7a–c). Control DLD1 cells showed a predominantly pseudo-diploid population (~80 %) (45–46 chromosomes) and another sub-population of ~20 % cells showing chromosomal losses and gains (Fig. S7d). An increase in the number of cells showing chromosomal losses (43–45 chromosomes) (*p* = 0.0345) and gains (47–48 chromosomes) was detected upon Lamin A/C Kd (Fig. S7a, b, d). Remarkably, Lamin B2 Kd showed a significant increase in chromosomal gains (47–49 chromosomes) (*p* = 0.018) (Fig. S7a, c, d). This is consistent with chromosomal aneuploidies induced in DLD1 cells upon Lamin B2 depletion (Kuga et al. 2014). Karyotype analyses from metaphase spreads derived from control, Lamin A/C, or B2 Kd cells did not reveal any consistent chromosomal translocations (Fig. S7a–c). Notably, a comparable level of CIN (~30 %) is associated with colorectal cancer initiation and progression (Bardi et al. 1995; Ried et al. 1996).

Taken together, these studies show a specific induction of chromosomal aneuploidies upon Lamin A/C and Lamin B2 depletion in DLD1 cells. We next examined the spatial organization of both the diploid and the aneuploid CTs in Lamin-depleted cells.

### Lamin A/C depletion does not perturb conserved positions of diploid and aneuploid chromosome territories

**Fig. 4:**
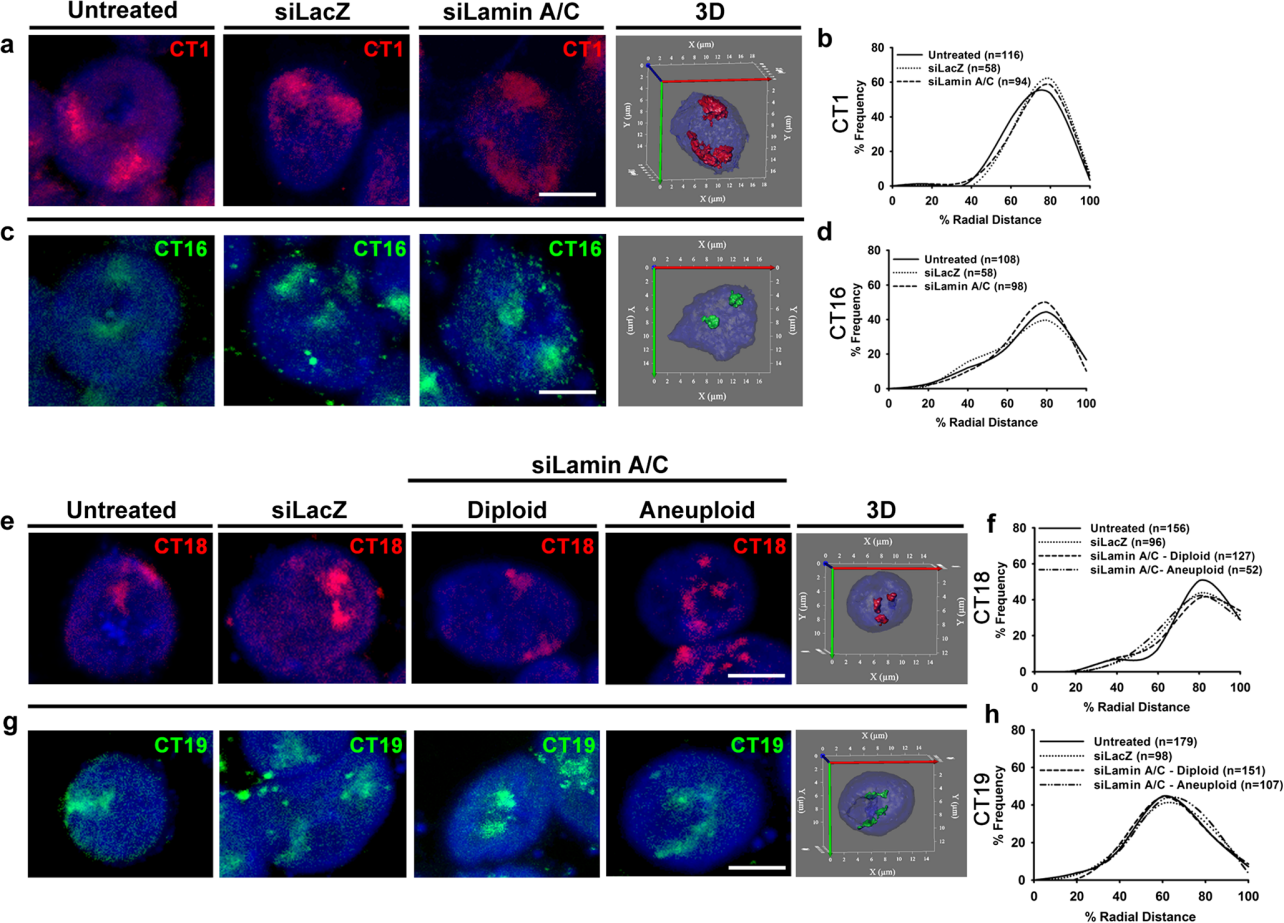
Diploid and aneuploid chromosome territories assume conserved positions upon Lamin A/C depletion. **a**–**d** Representative images and radial distance distribution profiles for 3D-FISH performed on untreated, siLacZ-treated, and siLamin A/C-treated DLD1 cells for CT1 (**a**, **b**) and CT16 (**c**, **d**). Each *panel* shows merged maximum intensity projections of a representative nucleus from control (untreated, siLacZ) and siLamin A/C cells with its corresponding 3D reconstruction (3D). **b**, **d** Representative radial distance distribution profiles (pooled across experiments) of CT1 (*N* = 1) and CT16 (*N* = 2) binned into five sub-shells of ~20 % radial distance each. In each replicate, radial distances were measured for ~50–100 CTs. No significant change was detected in the radial distance distribution profiles of either diploid CT1 or CT16 upon Lamin A/C Kd (*p* > 0.05). **e**–**h** Merged maximum intensity projections for 3D-FISH images and radial distance distribution profiles for CT18 (**e**, **f**) and CT19 (**g**, **h**) which show aneuploidy upon Lamin A/C Kd. Representative 3D reconstructions show aneuploidy upon Lamin A/C Kd. **f**, **h** Radial distance distribution profiles (pooled across experiments) of CT18 (*N* = 2) and CT19 (*N* = 3) binned into five sub-shells of ~20 % radial distance each. In each replicate, radial distances were measured for ~50–100 CTs. No significant changes were detected in the distribution profiles for CT18 and CT19 in Lamin A/C Kd diploid or aneuploid cells (*p* > 0.05). *Scale bar* ~5 μm. Fisher’s exact test and χ^2^ test was used to test statistical significance of distribution in different sub-shells of the nucleus. *N* number of independent experiments (biological replicates) contributing to the data, *n* number of chromosome territories

**Fig. 5:**
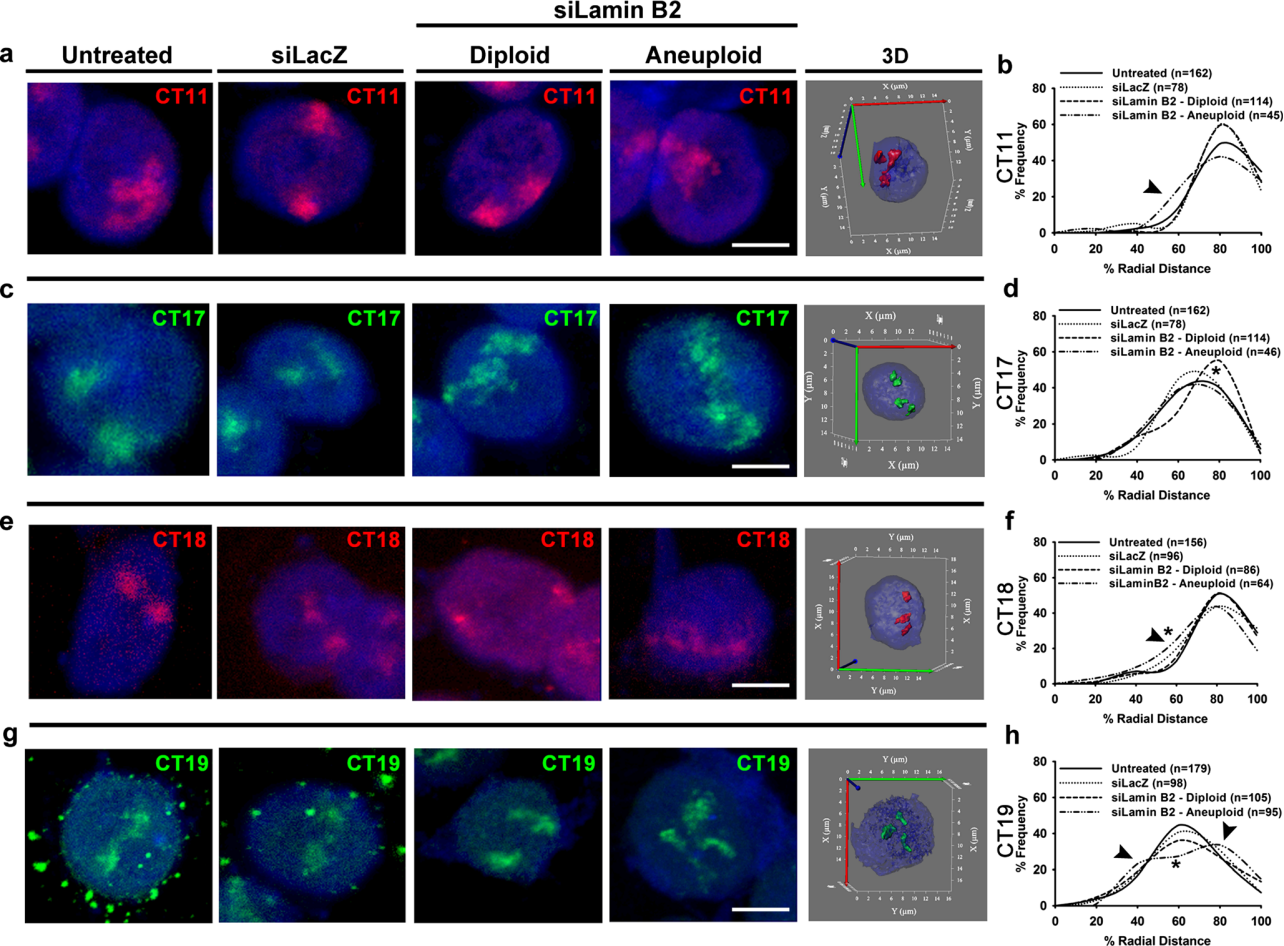
Aneuploid chromosome territories are mislocalized upon Lamin B2 depletion. Images from merged maximum intensity projection of 3D-FISH and radial distance distribution profiles for CT11 (**a**, **b**), CT17 (**c**, **d**), CT18 (**e**, **f**), and CT19 (**g**, **h**) which show aneuploidy upon Lamin B2 Kd. Representative 3D reconstructions show aneuploidy in the interphase nucleus upon Lamin B2 Kd. **b**, **d**, **f**, **h** Radial distance distribution profiles (pooled across experiments) of CT11 (*N* = 2), CT17 (*N* = 2), CT18 (*N* = 2), and CT19 (*N* = 3) binned into five sub-shells of ~20 % radial distance each. In each replicate, radial distances were measured for ~50–100 CTs. **b** Aneuploid CT11 shows an increase in the sub-population in shell III (RD ~60 %) over the control. **d** Diploid CT17 shows an increase in the sub-population in shell IV (RD ~80 %) over the control (*p* = 0.0145). **f** Aneuploid CT18 shows an increase in the sub-population in shell III (RD ~60 %) over the control (*p* = 0.0431). **h** Aneuploid CT19 showed a significantly different distribution of CT positions as compared to untreated cells (*p* = 0.0157). Specifically, it shows a decrease in the sub-population in shell III (RD ~60 %) over the control (*p* = 0.0061) and an increase in the subpopulations in shell II (RD ~40 %) and shell IV (RD ~80 %), respectively. *Scale bar* ~5 μm. Fisher’s exact and χ^2 ^test was used to test statistical significance of distribution in sub-shells of the nucleus. *N* number of independent experiments (biological replicates) contributing to the data, *n* number of chromosome territories. *Arrowheads*: mislocalized sub-populations of aneuploid chromosome territories upon Lamin B2 Kd

The spatial organization of chromosomes in the interphase nucleus largely correlates with gene density and, therefore, its expression levels, with gene-rich chromosomes in the nuclear interior being more transcriptionally active as against gene-poor chromosomes toward the nuclear periphery (Goetze et al. 2007). We performed 3D-FISH followed by confocal imaging and 3D radial distance measurements of diploid and aneuploid CTs in Lamin-depleted cells. The radial distance measurements have been represented both as a (i) dot scatter plots of raw data (Fig. S8) and (ii) binned into five shells of ~20 % of the nuclear sub-volume (0 %—nuclear center, 100 %—nuclear periphery) (Figs. 4 and 5). It is pertinent to reiterate that the innermost nuclear shell enriched in euchromatin is relatively more transcriptionally active as compared to the peripheral shell (~80–100 %) enriched in heterochromatin (Goetze et al. 2007). Three-dimensional FISH analyses of CT1 (gene density 15.83 genes/Mbp) and 16 (gene density 17.05 genes/Mbp) in Lamin A/C-depleted cells did not show a change in its radial distance distribution (*p* > 0.05) (Figs. 4a–d and S8a, b, Table 1). Furthermore, their relative positions in the nuclear sub-shells remained unchanged and conserved when compared to control cells (Fig. 4a–d). Radial distance measurements of CT18 (shell IV, RD ~80 %) and CT19 (shell III, RD ~60 %) in diploid or aneuploid cells remained conserved toward the nuclear periphery and nuclear interior, respectively (Figs. 4e–h and S8c, d). Radial distance measurements of CT1, 16, 18, and 19 in the siLacZ-treated DLD1 cells remained conserved as compared to untreated control cells (Fig. 4b, d, f, h). In summary, despite the specific depletion of Lamin A/C, the diploid or aneuploid gene-rich or gene-poor CTs showed a remarkable conservation in their spatial localization consistent with their gene densities.

**Table 1:** Median % radial distance (RD) and interquartile range (IQR) of chromosome territories upon Lamin A/C Kd in DLD1 cells

Chromosome no.	Gene density (genes/Mbp)	Untreated		siLacZ		siLamin A/C			
						Diploid		Aneuploid	
		Median	IQR	Median	IQR	Median	IQR	Median	IQR
CT1	15.83	62.27	14.11	65.49	14.98	66.07	19.17	–	–
CT16	17.05	67.42	25.91	64.3	25.31	62.85	22.88	–	–
CT18	8.21	73.63	18.20	72.5	23.61	73.73	22.24	71.14	26.08
CT19	37.08	52.08	27.02	51.73	23.44	54.86	23.8	51.45	23.64

### Lamin B2 depletion mislocalizes aneuploid chromosome territories

The increase in aneuploid cells upon Lamin B2 Kd is consistent with the known impact of Lamin B2 depletion in colorectal cancer cells (Kuga et al. 2014). We next analyzed the radial distance distributions of chromosomes that were transcriptionally deregulated upon Lamin B2 Kd in DLD1 cells. Although CT11 is gene rich (~17.5 genes/Mbp), it shows a peripheral (shell IV: RD ~80 %) nuclear localization in control cells (Fig. 5a, b). Analyses of radial distance distribution of CT11 in Lamin B2 Kd cells showed a conserved radial distance distribution in diploid cells (shell IV: RD ~80 %) (Fig. 5b). Aneuploid CT11 shows a mislocalized sub-population toward the nuclear interior to shell III: RD ~60 % (Fig. 5b). Interestingly, diploid CT17 (gene rich) showed a significant shift toward the nuclear periphery (shell IV: RD ~80 %) as compared to control cells (shell III: RD ~60 %) (Fig. 5c, d). However, the aneuploid CT17 does not show a mislocalization as compared to control cells and remains clustered in shell III (RD ~60 %) (Fig. 5d). It is plausible that the extra copy of CT17 may cluster into a single shell in Lamin B2 Kd cells, while one of the homologs in the diploid CT17 is mislocalized, suggesting differential effects that are exerted on CT17 in diploid and aneuploid Lamin B2 Kd cells—the functional relevance of which remains to be tested. The gene-poor and diploid CT18 in Lamin B2 Kd cells showed a predominantly peripheral nuclear localization (shell IV, RD ~80 %). In addition, aneuploid CT18 shows an increase in a sub-population of cells toward the nuclear interior (shell III, RD ~60 %) (Fig. 5e, f). In contrast, the gene-rich CT19 predominantly localized toward the nuclear interior (shell III, RD ~60 %) in control and diploid cells depleted in Lamin B2. However, aneuploid CT19 showed a striking mislocalization in its distribution into nuclear sub-shells, repositioning further toward the nuclear interior (shell II: RD ~40 %) and nuclear periphery (shell IV: RD ~80 %) (Fig. 5g, h). Radial distance measurements of CT11, 17, 18, and 19 in the siLacZ-treated DLD1 cells remained conserved as compared to untreated control cells (Fig. 4b, d, f, h). Taken together, aneuploid CTs show mislocalized sub-populations upon Lamin B2 depletion. We examined the interquartile range (IQR) of the radial distance distributions of the CTs, which consistently showed a greater IQR (22.73–31.19) for the aneuploid cells than for the diploid cells (11.21–29.97) (Table 2).

**Table 2:** Median % radial distance (RD) and interquartile range (IQR) of chromosome territories upon Lamin B2 Kd in DLD1 cells

Chromosome no.	Gene density (genes/Mbp)	Untreated		siLacZ		siLamin B2			
						Diploid		Aneuploid	
		Median	IQR	Median	IQR	Median	IQR	Median	IQR
CT11	17.5	76.19	17.25	73.55	16.81	75.06	11.21	73.11	22.73
CT17	24.21	57.94	24.53	57.79	18.84	62.22	14.69	57.6	27.87
CT18	8.21	73.63	18.20	72.5	23.61	70.45	19	69.25	23.3
CT19	37.08	52.08	27.02	51.73	23.44	53.88	29.97	57.23	31.19

### Spatial organization of gene loci is altered upon Lamin B2 depletion

**Fig. 6:**
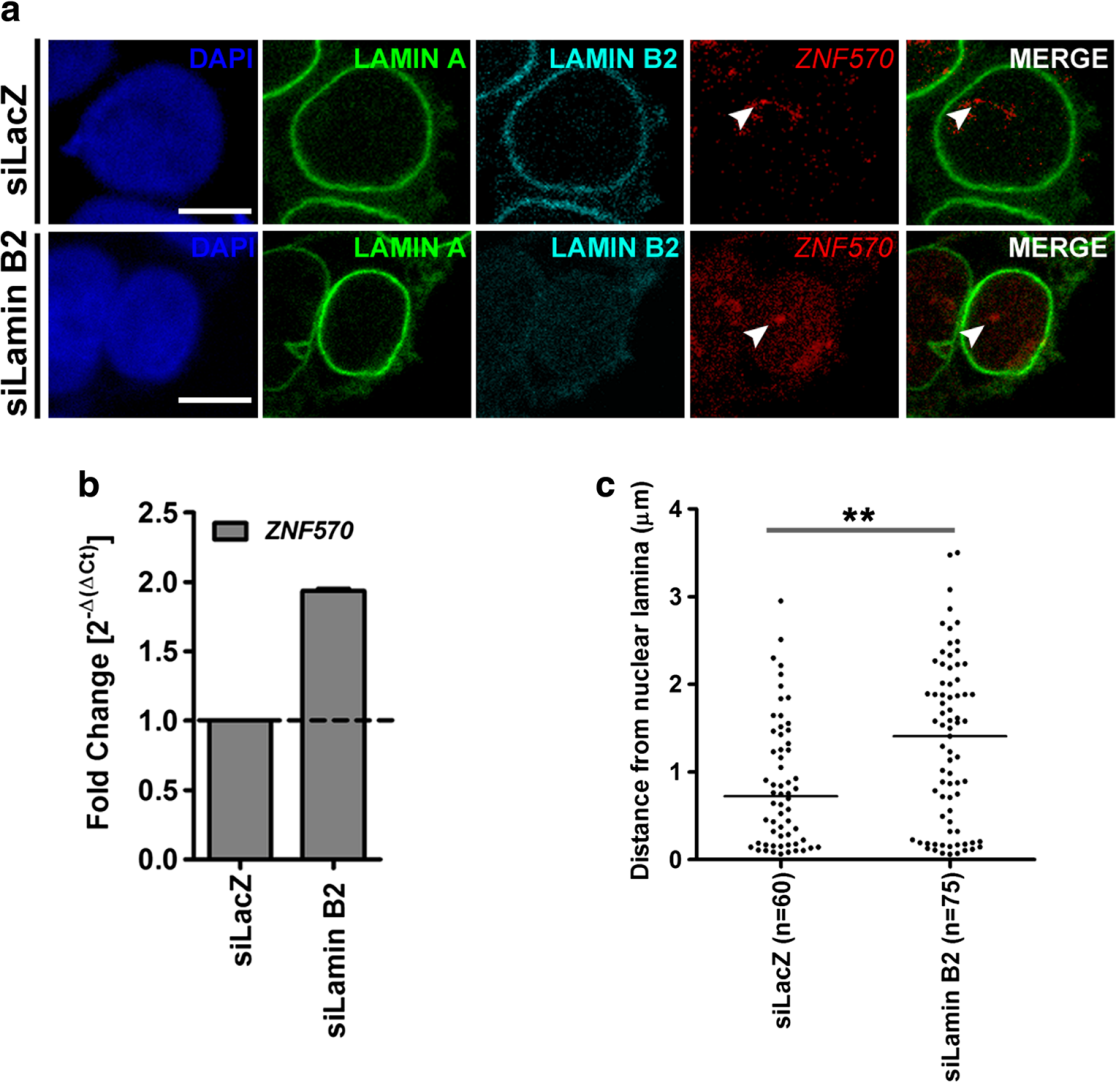
Enhanced expression level and altered spatial organization of gene loci upon Lamin B2 depletion. **a** Representative images (from a single optical section of a confocal image stack) of 3D Immuno-FISH for Lamin A (*green*), Lamin B2 (*cyan*), *ZNF570* (*red*), and DAPI (*blue*) performed on siLacZ- and siLamin B2-treated DLD1 cells. *Scale bar* ~5 μm. **b** qRT-PCR showing upregulation of *ZNF570* upon Lamin B2 Kd in DLD1 cells. Data shown is a representation out of two independent experiments *Error bars* represent SEM. **c** Dot scatter plot showing least distance between *ZNF570* locus and Lamin A signal in siLacZ- and siLamin B2-treated DLD1 cells, horizontal bar represents median. In each of siLacZ and siLamin B2 cells, ~60–70 gene loci signals were quantified. The distance of *ZNF570* locus with respect to the lamina was significantly greater in the siLamin B2 cells (*p* = 0.004, K-S test). *Arrowheads*: *ZNF570* gene locus

We show that Lamin B2 depletion mislocalizes aneuploid CTs. We therefore examined if the mislocalization of aneuploid CTs also impinges on the spatial organization and function of a target gene (Fig. 6). We examined the role of Lamin B2 in regulating gene expression of a candidate gene on chromosome 19. The spatial organization of gene loci in the interphase nucleus is non-random and correlates with its expression levels. Gene loci that are overexpressed typically loop-out of their respective CTs (Chambeyron and Bickmore 2004; Volpi et al. 2000). Furthermore, gene loci associate with nuclear landmarks such as the nuclear lamina, Polycomb bodies, which further impact its transcription status (Bracken et al. 2006; Guelen et al. 2008). The nuclear lamina is predominantly a transcriptionally repressive zone, and inactive genes typically associate with nuclear Lamins at the nuclear periphery in contrast to active genes (Guelen et al. 2008; Harr et al. 2015; Peric-Hupkes et al. 2010; Reddy et al. 2008).

We adopted the nuclear lamina as a reference for examining the spatial organization of the gene locus in 3D (Reddy et al. 2008; Zullo et al. 2012) (Fig. 6a). Since we consistently detected chromosome 19 aneuploidies upon Lamin B2 knockdown, we examined the spatial organization of a candidate gene locus *ZNF570* (Chr.19q13.12) (Fig. 6a). The *ZNF570* gene locus was also found to be aneuploid in ~25 % Lamin B2-depleted cells. Gene expression profiling using microarrays showed a ~2.96-fold upregulation of *ZNF570*, which was corroborated by qRT-PCR (~2-fold) (Fig. 6b). We measured the distance of the fluorescently labeled *ZNF570* gene locus with respect to the nuclear lamina (Lamin A) that we considered as the origin, i.e., 0 μm (Fig. 6c). The *ZNF570* gene locus showed a predominant position proximal to the nuclear lamina (median = 0.72 μm) in control cells (siLacZ) and showed a significant shift away from the nuclear lamina upon Lamin B2 depletion (median = 1.40 μm) (Fig. 6c). The spatial organization of *ZNF570* showed a similar repositioning away from the nuclear lamina in diploid and aneuploid Lamin B2 Kd cells (Fig. S9a, b). Furthermore, we detected a significant increase in the number of RNA transcript signals for *ZNF570* in Lamin B2-depleted cells (Fig. S9c, d). Thus, a loss of Lamin B2 repositions *ZNF570* gene locus (diploid and aneuploid) away from the nuclear lamina, which further manifests as an increase in transcript levels detected in single cells (RNA-FISH) as well as at the population level (qRT-PCR).

### Chromosome territory volumes are altered in Lamin-depleted cells

**Fig. 7:**
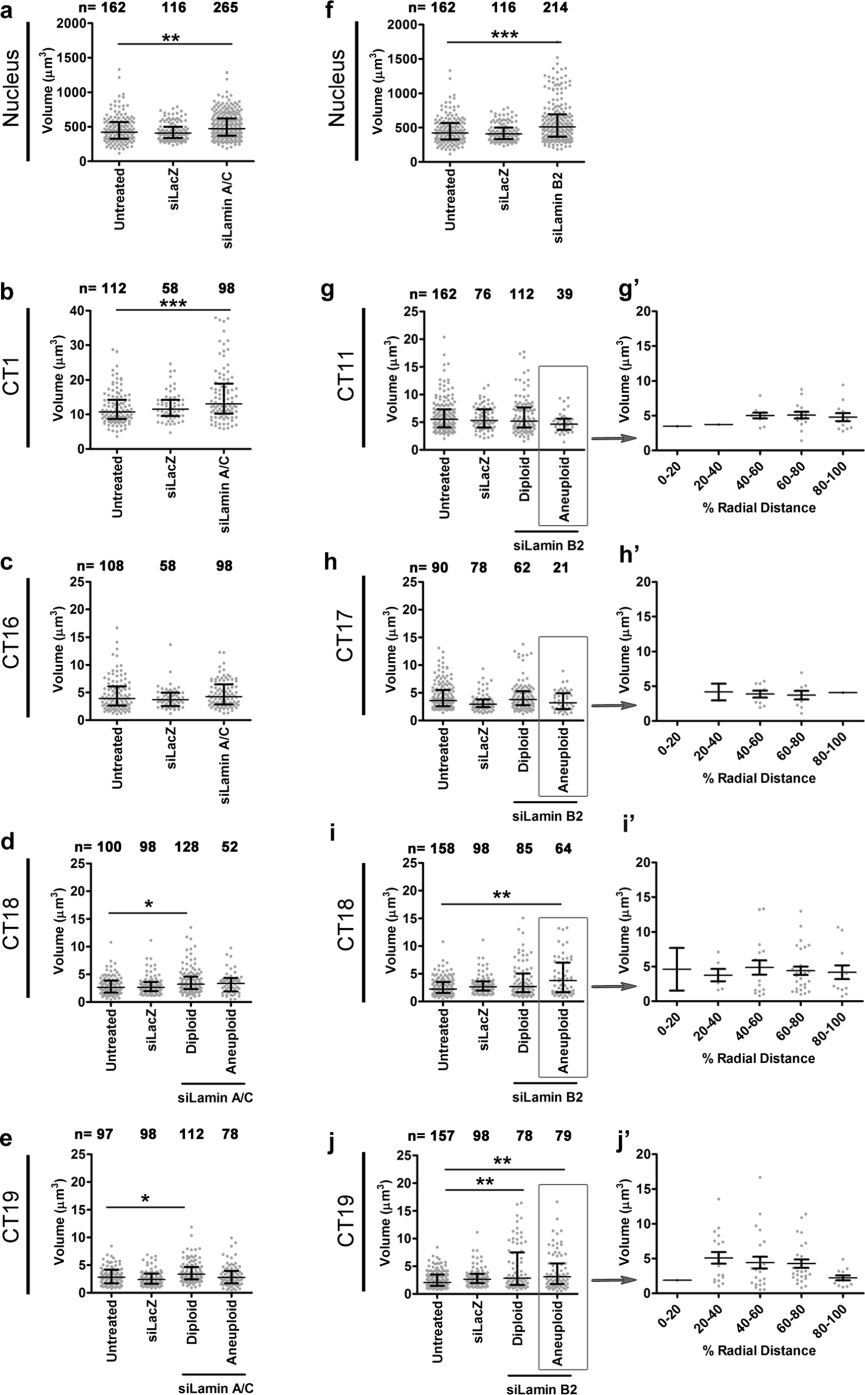
Chromosome territories show volume changes upon Lamin depletion. **a**–**j** Dot scatter plots for volumes of nuclei and chromosome territories upon Lamin A/C and Lamin B2 depletion in DLD1 cells. *Horizontal bars* indicate median with the interquartile range. **a** A significant increase was detected for the nuclear volume (~1.1-fold) upon Lamin A/C Kd (*p* = 0.0001), *N* = 5. **b** A significant increase was detected for volumes of CT1 (*p* = 0.0009), *N* = 1 upon Lamin A/C Kd. **c** No significant difference in volume was detected for CT16, *N* = 2. **d**, **e** A significant increase in the volume of diploid CT18 (*p* = 0.0149), *N* = 2, and CT19 (*p* < 0.0001), *N* = 2, while aneuploid CT18 and CT19 do not show a significant difference in volume upon Lamin A/C Kd. **f** Nuclear volume shows a significant increase upon Lamin B2 Kd (~1.2-fold, *p* < 0.0001), *N* = 5. **g**, **h** No significant difference in volume for either diploid or aneuploid CT11 (*N* = 2) or CT17 (*N* = 1) upon Lamin B2 Kd. **i** A significant increase in the volume of aneuploid CT18 (*p* = 0.0019), *N* = 2. **j** Significant increase in the volume of diploid and aneuploid CT19 upon Lamin B2 Kd (*p* = 0.0004), *N* = 2. **g**’–**j**’ Distribution of the volumes of aneuploid chromosome territories (CT11, CT17, CT18, and CT19) across nuclear sub-shells upon Lamin B2 depletion. No significant difference was detected in the volumes of aneuploid CT across nuclear sub-shells. Kruskal–Wallis test was used to test for statistical significance. *N* number of independent experiments (biological replicates) contributing to the data, *n* number of nuclei (**a**, **f**) or chromosome territories (**b**–**e**, **g**–**j**) scored

Lamin A mutation (E161K) showed a decrease in the volume of chromosome 13 territory in human fibroblasts (Mewborn et al. 2010). Volumes of chromosome 18 and 19 territories showed an increase in Lamin B1-depleted DLD1 cells (Camps et al. 2014). We next examined if Lamin A/C or B2 depletion affects the volumes of CTs in either diploid or aneuploid cells (Fig. 7, Tables 3 and 4). The nuclear volume showed a significant increase (~1.1-fold) upon Lamin A/C Kd (Fig. 7a). Diploid chromosome 1, 18, and 19 territories showed a significant increase in their volumes (~1.2-fold) upon Lamin A/C depletion (Fig. 7b, d, e). Chromosome 16 did not show a change in its volume (Fig. 7c). Aneuploid CT18 and CT19 did not show a significant change in volume upon Lamin A/C Kd (Fig. 7d, e). This suggests a role for Lamin A/C in either directly or indirectly regulating CT volumes, primarily of diploid CTs.

**Table 3:** Median volume of chromosome territories upon Lamin A/C Kd in DLD1 cells

Chromosome no.	Untreated (μm^3^)	siLacZ (μm^3^)	siLamin A/C (μm^3^)	
			Diploid	Aneuploid
CT1	10.73	11.51	13.05*	–
CT16	3.89	3.69	4.26	–
CT18	2.65	2.65	3.21*	3.36
CT19	2.83	2.39	3.37*	2.75

*Significant (*p* < 0.05)

**Table 4:** Median volume of chromosome territories upon Lamin B2 Kd in DLD1 cells

Chromosome no.	Untreated (μm^3^)	siLacZ (μm^3^)	siLamin B2 (μm^3^)	
			Diploid	Aneuploid
CT11	5.53	5.32	5.22	4.64
CT17	2.99	2.94	3.32	4.07
CT18	2.23	2.65	2.66	3.76*
CT19	2.06	2.65	2.84*	3.08*

*Significant (*p* < 0.05)

Lamin B2 depletion shows an ~1.2-fold increase in nuclear volume (Fig. 7f). CT11 and CT17 did not show a change in volume in either diploid or aneuploid state (Fig. 7g, h). CT18 showed an increase in volumes (~1.7-fold) in the aneuploid state, and CT19 showed an increase in volume in the diploid (~1.4-fold) as well as aneuploid state (~1.5-fold) (Fig. 7i, j). In summary, Lamin A/C and B2 depletion impacts nuclear volumes. While Lamin A/C Kd primarily affects volumes of diploid CTs, Lamin B2 Kd impacts volumes of mainly aneuploid CTs.

To further probe if mislocalized aneuploid CTs show an altered volume upon Lamin B2 Kd, we examined the volumes of aneuploid CTs across concentric nuclear sub-shells (Fig. 7g’–j’). We did not detect a significant difference in the volumes of aneuploid CTs whether they were localized toward the nuclear interior or toward the nuclear periphery, further suggesting that the altered volume of aneuploid CTs tested here (CT18, CT19) may not correlate with its mislocalization into a different nuclear sub-shell.

### Lamin depletion destabilizes spatial constraints that confine chromosome territories into distinct nuclear sub-volumes

**Fig. 8:**
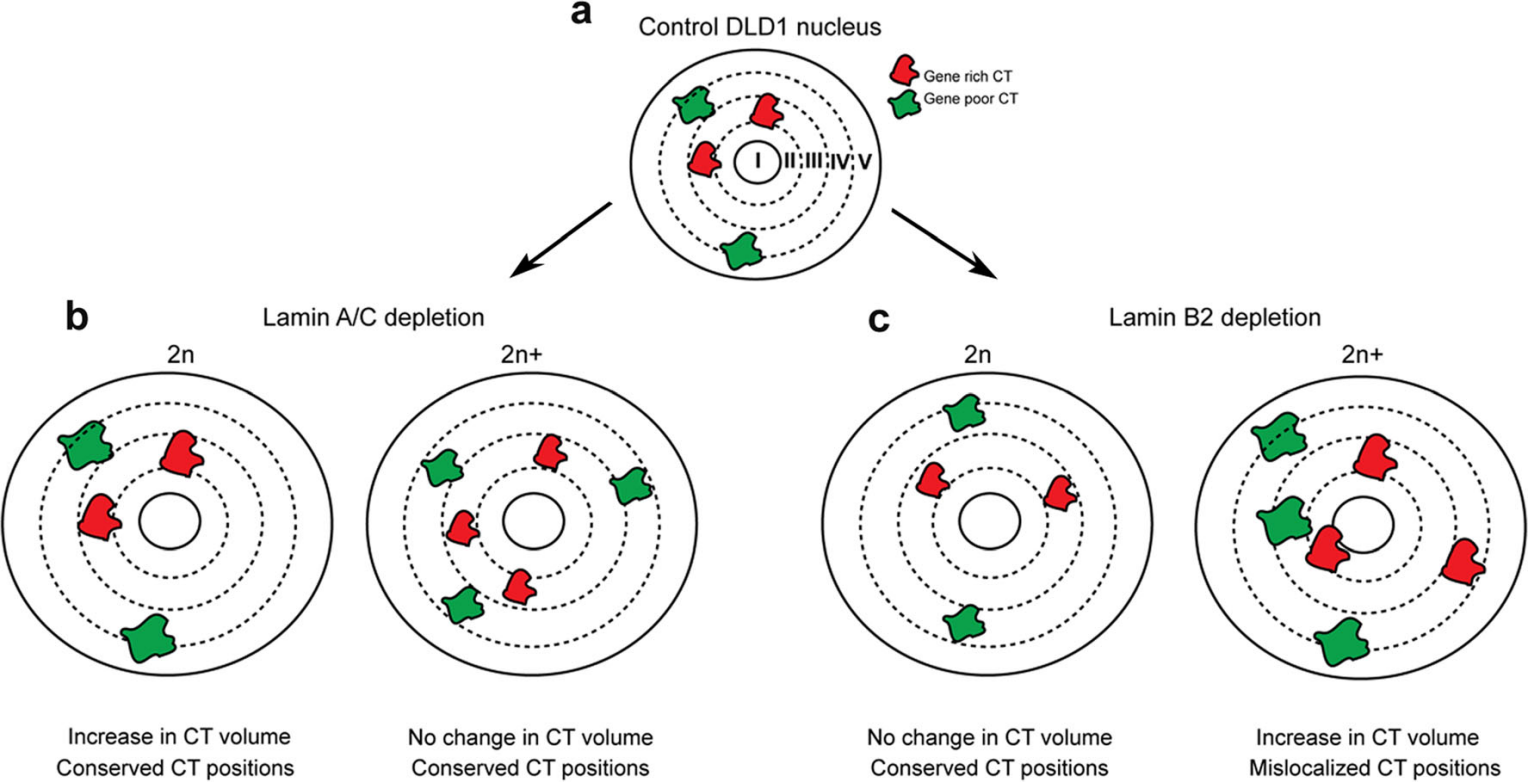
Lamin B2 depletion perturbs positioning constraints of aneuploid chromosome territories in the interphase nucleus. **a** Schematic representation of the nucleus divided into five concentric sub-shells (I–V) of ~20 % radial distance. Gene-rich CT occupies the nuclear interior (sub-shell: III), while gene-poor CT occupies the nuclear periphery (sub-shell: IV). Nuclear volume shows an increase in both Lamin A/C and Lamin B2 Kd. **b** Representation of the diploid (2n) and the aneuploid (2n+) nucleus upon Lamin A/C Kd. Both diploid and aneuploid CTs show conserved positions, while diploid CTs show a significant increase in volume upon Lamin A/C Kd. **c** Representation of the diploid (2n) and the aneuploid (2n+) nucleus upon Lamin B2 Kd. Diploid CTs show conserved chromosome positions and no change in volume, while aneuploid CTs show mislocalization of CTs and significant change in volume upon Lamin B2 Kd

We examined the spatial organization of both diploid and aneuploid CTs in Lamin-depleted cells. Lamin depletion showed an increase in nuclear volume of ~1.1-fold in Lamin A/C Kd cells and ~1.2-fold in Lamin B2 Kd cells. A spherical nucleus can be grossly sub-divided into five concentric sub-shells, with the gene-rich CTs predominantly toward the nuclear interior (shell II–III), while gene-poor CTs are toward the nuclear periphery (shell IV–V) (Fig. 8a). Chromosomal aneuploidies generated upon Lamin A/C and B2 depletion generate diploid and aneuploid chromosomal sub-populations. Diploid CTs in either Lamin A/C- or B2-depleted cells show conserved positions in a gene-density-dependent manner. However, Lamin A/C knockdown cells showed an increase in CT volume in the diploid sub-population of cells (Fig. 8b). In contrast, aneuploid CTs in Lamin B2-depleted cells are strikingly mislocalized and show altered volumes in the interphase nucleus (Fig. 8c). Our results strongly implicate the presence of additional underlying Lamin B2-dependent mechanisms that facilitate gene-density-based chromosome positioning in the interphase nucleus. This potentially includes (i) direct associations of Lamin B2 with chromatin and (ii) protein–protein interactomes of Lamin B2 or a combination of both these factors. Taken together, our studies highlight a unique role for Lamin B2 in regulating the spatial localization of aneuploid CTs in the interphase nucleus.

## Discussion

Lamins are required for the maintenance of nuclear structure and function. Furthermore, Lamins are necessary for the appropriate localization of CTs in the interphase nucleus (Malhas et al. 2007; Mewborn et al. 2010; Shimi et al. 2008). Our results suggest a requirement for Lamin B2 since its absence results in mislocalization of aneuploid CTs. The specific role of Lamin B2 in regulating chromosomal ploidy and chromosome positioning is difficult to uncouple. While both A- and B-type Lamins are involved in regulating spindle assembly during mitosis (Goodman et al. 2010; Kuga et al. 2014; Qi et al. 2015; Tsai et al. 2006), Lamins are primarily structural proteins that maintain the spatial organization of the genome (Peric-Hupkes and van Steensel 2010). While both Lamin A/C and Lamin B2 depletion induced CIN, primarily Lamin B2 depletion resulted in a mislocalization of aneuploid CTs. There are conflicting reports on the expression levels of Lamin A/C in colorectal cancers (Belt et al. 2011; Foster et al. 2011; Moss et al. 1999; Willis et al. 2008; Wu et al. 2009). In contrast, Lamin B2 was shown to be a differentially expressed protein between colorectal cancers with and without CIN—with increase in Lamin B2 expression conferring protection against CIN (Kuga et al. 2014). It is noteworthy from our results that Chr.17, 18, and 19 (from the specific subset of chromosomes that we examined) were consistently gained independently upon Lamin A/C or Lamin B2 depletion (Fig. S4i). This is suggestive of a pattern of chromosomal acquisitions potentially relevant for colorectal cancer progression (Tagawa et al. 1996). It remains to be examined if the extent of Lamin stoichiometry correlates with the extent of these chromosomal gains during colorectal cancer progression (Belt et al. 2011; Roth et al. 2010).

Our study highlights an interesting divergence of roles for Lamin A/C and Lamin B2. The role of Lamin B2 in maintenance of genome organization is rather underappreciated. Diploid DLD1 cells robustly express Lamins A/C, B1, and B2 that potentially compensate for one another in single Lamin knockdowns in order to maintain conserved chromosome positions. This is further reiterated in Lamin B1-depleted DLD1 cells that show a conserved localization of CT18 at the nuclear periphery (Camps et al. 2014). However, in aneuploid cells, an optimum level of Lamin B2 is a likely prerequisite for the correct spatial positioning of CTs. It is plausible that regulation of ploidy and positioning of aneuploid chromosomes are co-regulated by Lamin B2 through its commonly regulated interactors that function during both mitosis and interphase. One such candidate is the inner nuclear membrane protein SUN1 identified in a screen by Kuga et al. (2014) which associates with Lamin B2 during mitosis. However, the precise roles of Lamin B2-associated interactors in the context of maintaining the spatial organization of the genome is unclear.

Previous studies suggest a remarkable conservation in gene-density-based chromosome positioning patterns of even aneuploid chromosomes in the interphase nucleus in cancer cells (Cremer et al. 2003). A recent study on primary amniocyte cells shows that naturally aneuploid human Chr.18 and 21 territories assume conserved nuclear locations (Hervé et al. 2016). Further, artificially introduced gene-poor human Chr.7 or 18 and gene-rich Chr.19 into otherwise diploid DLD1 cells showed a gene-density-based conservation in their positioning patterns toward the nuclear periphery and nuclear interior, respectively (Sengupta et al. 2007). It is therefore likely that Lamins play a crucial role in the conservation of spatial organization of aneuploid (trisomies) CTs. It is conceivable that Lamin depletion destabilizes different subsets of Lamin interaction networks at the (i) nuclear periphery (linker of nucleoskeleton and cytoskeleton (LINC) complex), (ii) heterochromatin, (iii) nuclear interior, and (iv) perinucleolar heterochromatin (Dechat et al. 2010; Gruenbaum and Medalia 2015). At the nuclear periphery, Lamins interact with LEM-D proteins, LBR, and HP1 involved in heterochromatin organization (Clements et al. 2000; Foisner and Gerace 1993; Smith and Blobel 1994; Solovei et al. 2013). We surmise that Lamin B2 depletion results in a general weakening of the nuclear periphery due to a loss of its association with Lamin A/C and other factors such as LINC complex, LBR (Moir et al. 2000; Shimi et al. 2015; Ye et al. 1997). This not only maintains the integrity of the nuclear periphery but also tethers gene-poor CTs since aneuploid CT18 (in Lamin B2-depleted cells) disengages from the nuclear lamina and moves further into the nuclear interior. In the nucleoplasm, Lamins associate with chromatin organizers—CTCF, BANF1, and Lamina-associated polypeptide (LAP2α/β) that assist chromatin organization (Dechat et al. 2000; Gesson et al. 2016; Yusufzai et al. 2004). This is likely to impinge on the spatial organization of gene-rich CTs such as CT19 toward the nuclear interior. The role of phosphorylated Lamin A (in the nuclear interior as demonstrated in HeLa cells) (Kochin et al. 2014) in genome organization is unclear. Lamin A/C and LBR are required for tethering heterochromatin, while its absence results in a mislocalization of heterochromatin to the nuclear interior and, therefore, an inversion in nuclear architecture in murine rod cells (Solovei et al. 2009, 2013). Our studies suggest a similar alteration in genome organization as evidenced by the mislocalization of the gene-rich CT19 toward the nuclear periphery and gene-poor CT18 toward the nuclear interior specifically in Lamin B2-depleted aneuploid cells (Fig. 5f, h). This suggests a requirement specifically for Lamin B2 and its interactome in maintaining conserved CT positions. A careful biochemical analysis of these nuclear sub-fractions may provide further insights on these potential interactions with Lamin B2 required for the correct maintenance of CTs in a manner consistent with gene density. Alternatively, the decision to place chromosomes in unique nuclear locations is likely to be initiated during mitosis, where Lamins regulate chromosome segregation (Martin et al. 2010).

It is pertinent to note that Lamin-depletion-induced volume changes of CTs may further impinge on the spatial constraints that position CTs and their transcription potential (Fig. 7). However, our studies reveal that the volumes of aneuploid CTs are comparable irrespective of the nuclear space (sub-shells) that they occupy (Fig. 7). Notably, diploid CTs showed an increase in volume upon Lamin A/C Kd (Fig. 7). Gene expression profiling showed a downregulation of *SMC1A* (~2-fold) upon Lamin A/C but not in Lamin B2 knockdown. SMC1A regulates chromatin architecture and could potentially impinge on volumes of CTs upon Lamin A/C depletion (Phillips-Cremins et al. 2013). Essentially, our data suggests that maintenance of CT position and function is not just dependent on gene density but additionally requires Lamins and its interactors to function as the “zip code” for the correct placement and regulation of transcriptional activity of either diploid or aneuploid CTs in the interphase nucleus.

The altered position of the aneuploid chromosomes could potentially have a bearing on their interaction with the nuclear lamina and with neighboring CTs (Branco and Pombo 2006). Haploid and diploid KBM7 cells show variability in their LAD profiles, suggesting that LAD profiles are modulated by the ploidy of cells (Kind et al. 2015). It would be useful to compare LAD profiles in diploid versus aneuploid Lamin-depleted cells, although a potential technical limitation is to specifically enrich number of cells with chromosomal aneuploidies. In addition, the identification of chromatin contacts in single cells by Hi-C studies and RNA-Seq analyses would enable assessing the impact of chromosomal aneuploidies on chromatin contact frequencies and transcription (Hashimshony et al. 2012; Islam et al. 2012; Nagano et al. 2013).

Whole chromosomal gains in colorectal cancer cell lines and patient samples positively correlate with gene expression levels (Grade et al. 2007). Gene expression profiling of human Chr.5 and Chr.7 in colorectal cancer cells (HCT116 and DLD1 cells, respectively), Chr.13 trisomy (Edward’s syndrome), and Chr.21 trisomy in Down’s syndrome showed an upregulation of transcripts from the aneuploid chromosomes by ~1.1–1.5-fold (FitzPatrick et al. 2002; Mao et al. 2003; Stingele et al. 2012; Upender et al. 2004). Lamin depletion followed by gene expression profiling of Lamin A/C and B2 Kd DLD1 cells showed an ~1 % deregulation of the transcriptome in a non-overlapping manner (Fig. 2a). It remains to be examined if aneuploid chromosomes are transcriptionally active even when they are mislocalized in the interphase nucleus in Lamin-depleted cells.

Relatively fewer studies have previously examined the correlation between the spatial organization and expression status of gene loci in aneuploid cells. Amplified *C*-*MYC* (Chr.8q24) gene loci are localized external to chromosome 8 territory in colon cancer cell line HT-29 (Harnicarova et al. 2006). Interestingly, these loci were found to be transcriptionally active. The lamina is a transcriptionally repressive zone, and repositioning of gene loci away from the nuclear lamina is associated with an increase in its gene expression levels (Peric-Hupkes et al. 2010; Shachar et al. 2015), as shown for artificially targeted reporter genes (LacO gene visualized using LacI-GFP) in single cells (Finlan et al. 2008; Reddy et al. 2008) as well as an endogenous gene *TCRB*, in contact with the nuclear Lamina (Schlimgen et al. 2008; Shachar et al. 2015). Our studies reveal altered spatial organization of gene loci consistent with an increase in its expression levels in Lamin B2-depleted cells. The candidate gene locus *ZNF570* was not only repositioned away from the nuclear lamina in both diploid and aneuploid cells but also showed a significant increase in transcript signals in Lamin B2-depleted cells (Figs. 6 and S9).

Taken together, our studies suggest that chromosomal aneuploidies induced in Lamin-deficient cells may achieve enhanced transcriptional deregulation by sampling diverse sub-nuclear microenvironments in the interphase nucleus. Conversely, the presence of Lamins serves to not only prevent chromosomal aneuploidies but also dampen their spatial excursions in the nucleus. A thorough understanding of transcriptional outputs of aneuploid chromosomes in the context of their spatial organization would undoubtedly have far-reaching consequences on the mechanisms of cancer initiation and diseases associated with chromosomal aneuploidies.

## Electronic supplementary material

Below is the link to the electronic supplementary material.

Fig. S1 Lamin knockdowns do not perturb the expression of other lamins in DLD1 cells. **a** Viability of DLD1 cells was assayed in Lamin A/C, Lamin B1 and Lamin B2 Kd, using Trypan Blue uptake and MTT methods. Controls: untreated cells, siNeg (non-targeting siRNA) and *PLK1* Kd (Polo like Kinase1). *N* = number of biological replicates. Data shown is pooled from 3 independent biological replicates. **b** The extent of Lamin B1 Kd is ~60 % in DLD1 cells **c**-**d** Full blots showing the levels of Lamin A/C, and B2 upon Lamin A/C, Lamin B1 and Lamin B2 knockdowns respectively in DLD1 cells. Controls used are untreated cells, transfection with non-targeting siRNA (siLacZ). Loading control: Actin. (GIF 66 kb)

High Resolution Image (TIF 586 kb)

Fig. S2 Nuclear aberrations are induced upon Lamin A/C or B2 depletion. **a** Lamin A/C and Lamin B2 Kd show nuclear aberrations – invaginations, irregular nuclei, blebs and furrows. Scale bar ~ 5 μm. **b** frequency of nuclear aberrations increases upon Lamin A/C or Lamin B2 Kd. (GIF 116 kb)

High Resolution Image (TIF 1612 kb)

Fig. S3 qRT-PCR validation of candidate genes from whole genome expression arrays. **a** The whole genome expression data was validated using SyBr Green based qRT-PCR in Lamin A/C Kd and **b** Lamin B2 Kd respectively. A set of 12 candidate genes was selected for validation in Lamin A/C Kd and 6 genes in Lamin B2 Kd. Two unique qRT-PCR primers were designed for the candidate genes - (a) oligonucleotide from a feature on the array (Primer Set A) and (b) oligonucleotide of the gene not represented on the array (Primer Set B) (Table S1). All of the candidate genes show a correlation in the same general direction (fold change) as that of genome wide expression data. All qRT-PCR assays were performed in three independent biological replicates, each containing 3 technical replicates normalized to expression of *ACTIN*. Error bars represent SEM. (GIF 25 kb)

High Resolution Image (TIF 263 kb)

Fig. S4 Ploidy of candidate chromosomes upon Lamin depletion in the interphase nucleus. **a**-**d** Copy numbers of chromosomes **a** CT1, **b** CT16, **c** CT18 and **d** CT19 in interphase nuclei upon Lamin A/C depletion in DLD1 cells. Chromosomes 18 and 19 show aneuploidy upon Lamin A/C depletion. **e**-**h** Copy numbers of chromosomes **e** CT11, **f** CT17, **g** CT18 and **h** CT19 in interphase nuclei upon Lamin B2 depletion in DLD1 cells. Chromosomes 11, 17, 18 and 19 are aneuploid upon Lamin B2 depletion. **i** Plot showing extent of gains and losses of chromosomes 1,11,16,17,18 and 19 upon siLacZ, siLamin A/C and siLamin B2 treatments. ~30-50 nuclei were scored in each set. **j**-**k** Copy numbers of chromosomes **j** CT18 and **k** CT19 in interphase cells upon transfection with Lamin B2 scrambled oligo, which does not show aneuploidy. (GIF 65 kb)

High Resolution Image (TIF 537 kb)

Fig. S5 Immuno-FISH for Lamin A-CT18 and Lamin B2-CT19 upon Lamin depletion in DLD1 cells. **a** Immuno-FISH for Lamin A (green), CT18 (red) in control and Lamin A/C Kd cells. Scale bar ~ 10 μm. **b** quantification of fluorescence intensities of Lamin A in control and Lamin A/C Kd diploid and aneuploid cells **c** Immuno-FISH for Lamin B2 (green), CT19 (red) in control and Lamin B2 Kd cells. Scale bar ~ 10 μm. **d** quantification of fluorescence intensities of Lamin B2 in control and Lamin B2 Kd diploid and aneuploid cells. Comparable level of depletion of Lamin A and B2 was achieved in the diploid and aneuploid nuclei upon Lamin knockdown. (GIF 56 kb)

High Resolution Image (TIF 643 kb)

Fig. S6 Lamin knockdowns do not alter cell cycle profiles. **a**-**d** FACS profiles do not reveal significant changes in overall ploidy levels for **a** control (untreated), **b** siLamin B1 **c** siLamin A/C **d** siLamin B2 determined using Propidium Iodide staining. **e** Western Blots showing expression of Chk1 and Chk2 in two biological replicates upon knockdown of Lamin A/C and Lamin B2 in DLD1 cells. No difference in the levels of Chk1 or Chk2 was detected either upon Lamin A/C or B2 depletion. **f** Western Blots showing expression of pChk1 and pChk2 in two biological replicates upon knockdown of Lamin A/C and Lamin B2 in DLD1 cells. No difference in the levels of pChk1 was detected either upon Lamin A/C or B2 depletion, while pChk2 was hardly detectable. GAPDH was used as a loading control for **e** and **f**. (GIF 46 kb)

High Resolution Image (TIF 418 kb)

Fig. S7 Chromosomal gains and losses upon Lamin A/C, B2 Kd in DLD1 cells. **a**-**c** Representative images of inverted DAPI stained metaphase spreads in **a** control **b** siLamin A/C **c** siLamin B2 in DLD1 cells. Scale bar ~ 10 μm. Chromosomes were counted from 50–70 individual metaphase spreads derived from control, Lamin A/C Kd and Lamin B2 Kd cells. **d** Chromosome losses and gains in DLD1 cells enumerated by counting the number of DAPI stained chromosomes in metaphase spreads from control, Lamin A/C Kd and Lamin B2 Kd. The counts were classified as 45–46 (pseudo-diploid), <45 (losses) and >46 (gains). siLamin A/C: Significant increase in cells with chromosomal losses (p = 0.0345) and significant decrease (p = 0.0047) in the pseudo-diploid population. siLamin B2: significant increase in chromosomal gains (p = 0.018). (GIF 53 kb)

High Resolution Image (TIF 660 kb)

Fig. S8 Raw data showing % radial distances of chromosome territories upon Lamin depletion experiment-wise. **a**-**d** Each plot represents the radial distance of CT for each independent experiment **a** CT1 **b** CT16 **c** CT18 **d** CT19 upon siLamin A/C. **e**-**h** Each plot represents the radial distance of CT for each independent experiment **e** CT11 **f** CT17 **g** CT18 **h** CT19 upon siLamin B2. Horizontal bars in the dot scatter plot represent the medians. *n* = number of chromosome territories quantified. (GIF 91 kb)

High Resolution Image (TIF 657 kb)

Fig. S9 Increase in ZNF570 transcript signals upon Lamin B2 Kd. **a** Distance of ZNF570 gene locus from the lamina in diploid and aneuploid nuclei upon Lamin B2 Kd. **b** Distance of ZNF570 from the nuclear lamina plotted in bins of 0.5 μm each from siLacZ, siLamin B2 (diploid and aneuploid cells). Both diploid and aneuploid cells show repositioning of *ZNF570* away from the nuclear lamina. **c** representative RNA-FISH images for *ZNF570* (red) in control and siLaminB2 cells. **d** Quantification of the number of RNA-FISH signals upon total number of cells scored in control and siLamin B2 cells shows an increase in proportion of RNA signals upon siLamin B2. Data compiled from a single experiment. Arrowheads: RNA signals for *ZNF570*. (GIF 740 kb)

High Resolution Image (TIF 740 kb)

Table S1 List of qRT-PCR primers used in this study (DOCX 16 kb)
