# Supplementary figures and images for: Chromosomal aneuploidies induced upon Lamin B2 depletion are mislocalized in the interphase nucleus
Source: Chromosoma. 2016 Feb 27;126(2):223–44. doi: 10.1007/s00412-016-0580-y (PMC5371638; doi:10.1007/s00412-016-0580-y)

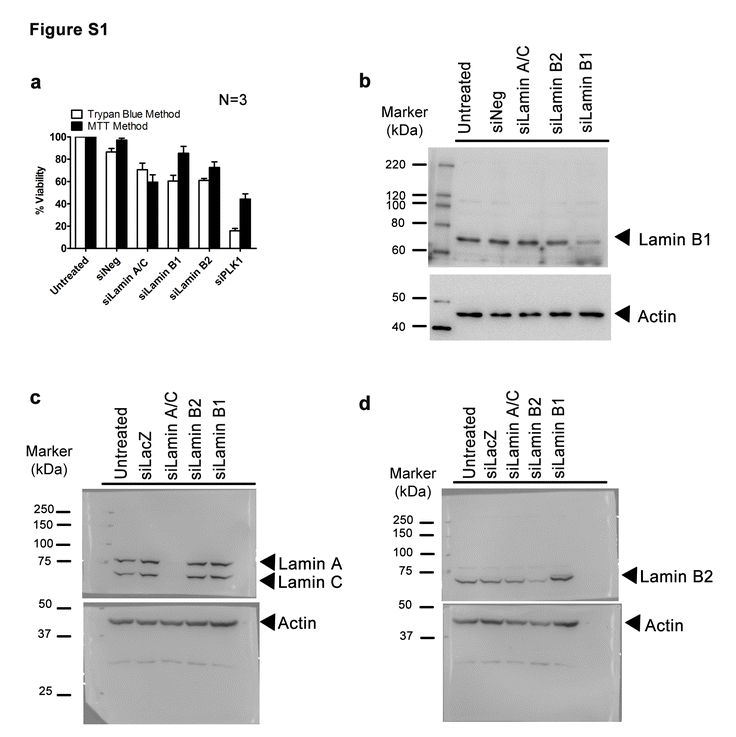

Supplement: Supplementary file 1 — Lamin knockdowns do not perturb the expression of other lamins in DLD1 cells. a Viability of DLD1 cells was assayed in Lamin A/C, Lamin B1 and Lamin B2 Kd, using Trypan Blue uptake and MTT methods. Controls: untreated cells, siNeg (non-targeting siRNA) and PLK1 Kd (Polo like Kinase1). N = number of biological replicates. Data shown is pooled from 3 independent biological replicates. b The extent of Lamin B1 Kd is ~60 % in DLD1 cells c-d Full blots showing the levels of Lamin A/C, and B2 upon Lamin A/C, Lamin B1 and Lamin B2 knockdowns respectively in DLD1 cells. Controls used are untreated cells, transfection with non-targeting siRNA (siLacZ). Loading control: Actin. (GIF 66 kb) [file 412_2016_580_Fig10_ESM.gif]

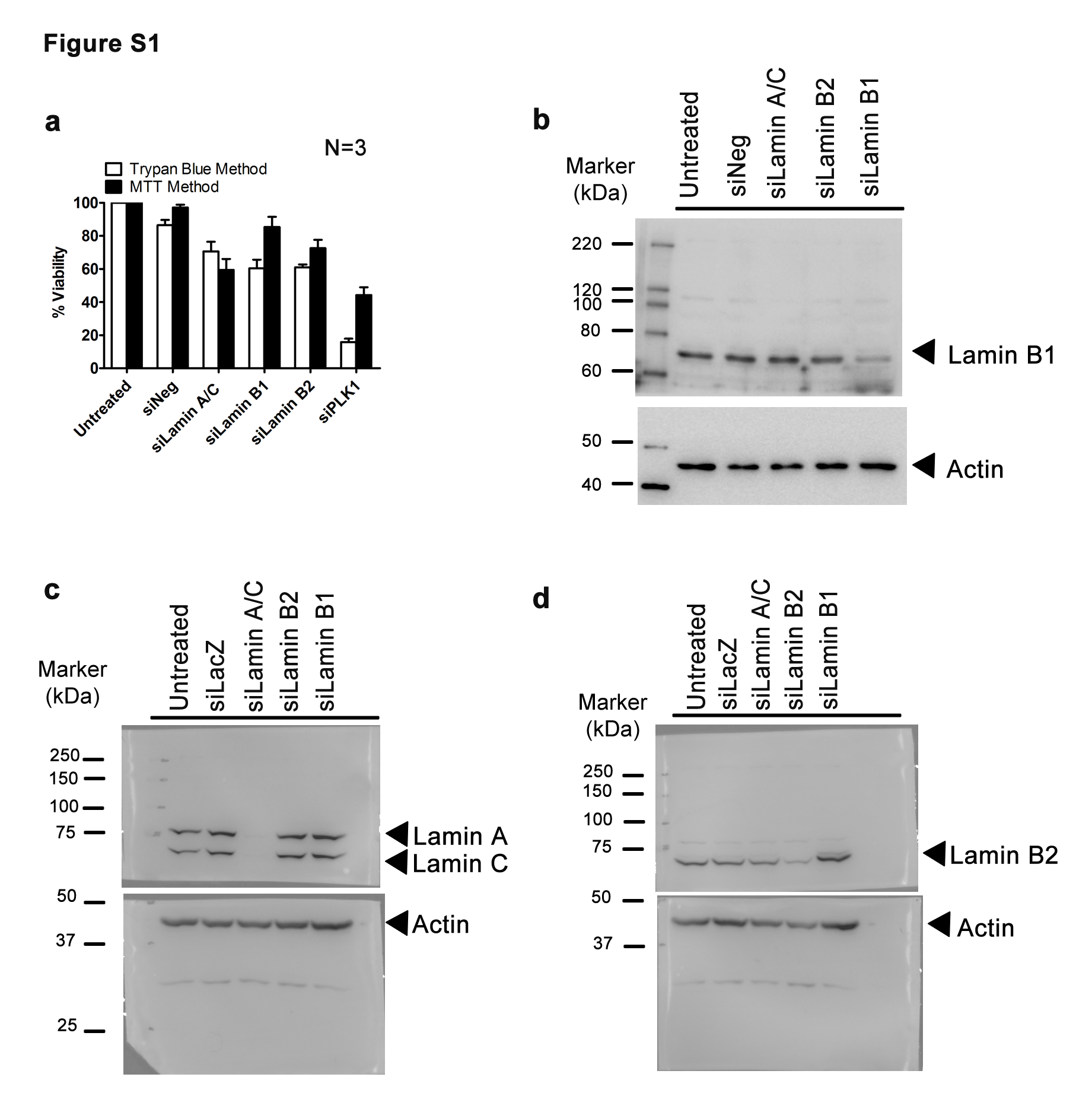

Supplement: Supplementary file 2 — High Resolution Image (TIF 586 kb) [file 412_2016_580_MOESM1_ESM.tif]

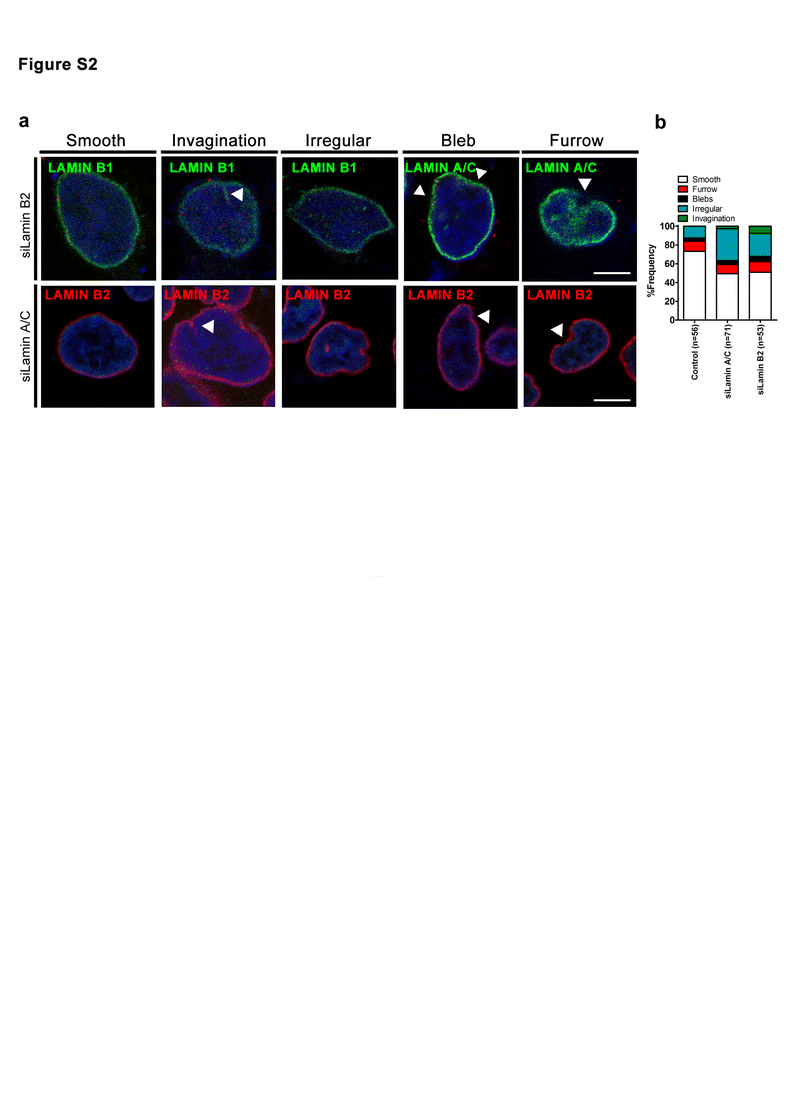

Supplement: Supplementary file 3 — Nuclear aberrations are induced upon Lamin A/C or B2 depletion. a Lamin A/C and Lamin B2 Kd show nuclear aberrations – invaginations, irregular nuclei, blebs and furrows. Scale bar ~ 5 μm. b frequency of nuclear aberrations increases upon Lamin A/C or Lamin B2 Kd. (GIF 116 kb) [file 412_2016_580_Fig11_ESM.gif]

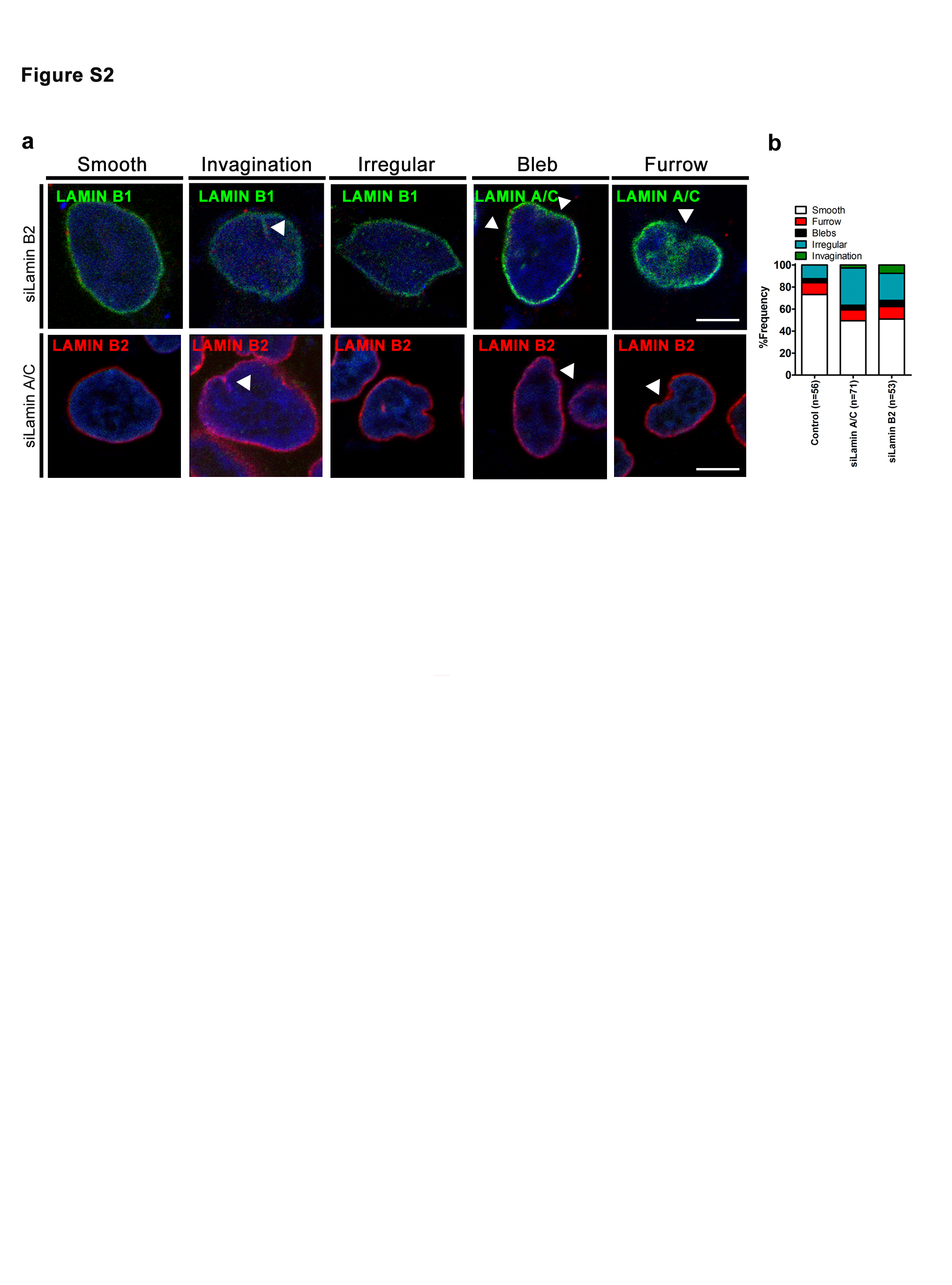

Supplement: Supplementary file 4 — High Resolution Image (TIF 1612 kb) [file 412_2016_580_MOESM2_ESM.tif]

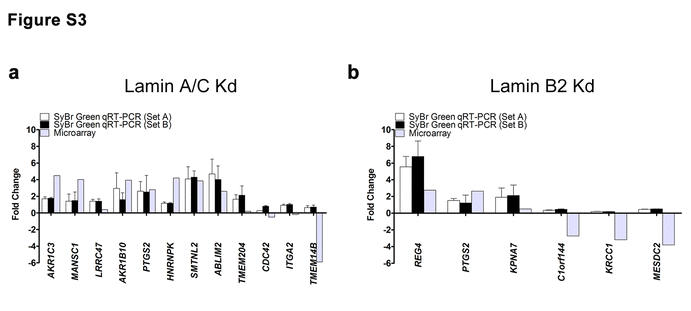

Supplement: Supplementary file 5 — qRT-PCR validation of candidate genes from whole genome expression arrays. a The whole genome expression data was validated using SyBr Green based qRT-PCR in Lamin A/C Kd and b Lamin B2 Kd respectively. A set of 12 candidate genes was selected for validation in Lamin A/C Kd and 6 genes in Lamin B2 Kd. Two unique qRT-PCR primers were designed for the candidate genes - (a) oligonucleotide from a feature on the array (Primer Set A) and (b) oligonucleotide of the gene not represented on the array (Primer Set B) (Table S1). All of the candidate genes show a correlation in the same general direction (fold change) as that of genome wide expression data. All qRT-PCR assays were performed in three independent biological replicates, each containing 3 technical replicates normalized to expression of ACTIN. Error bars represent SEM. (GIF 25 kb) [file 412_2016_580_Fig12_ESM.gif]

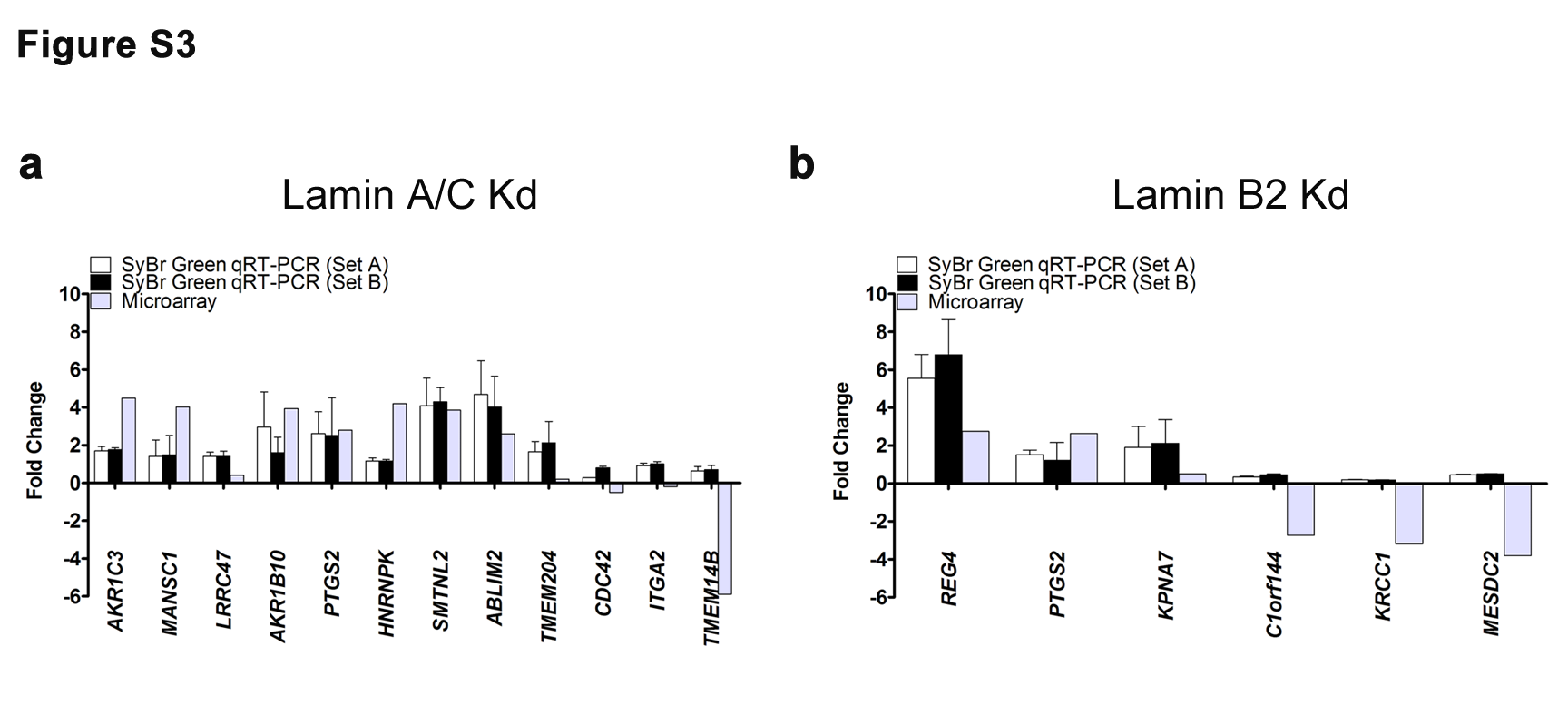

Supplement: Supplementary file 6 — High Resolution Image (TIF 263 kb) [file 412_2016_580_MOESM3_ESM.tif]

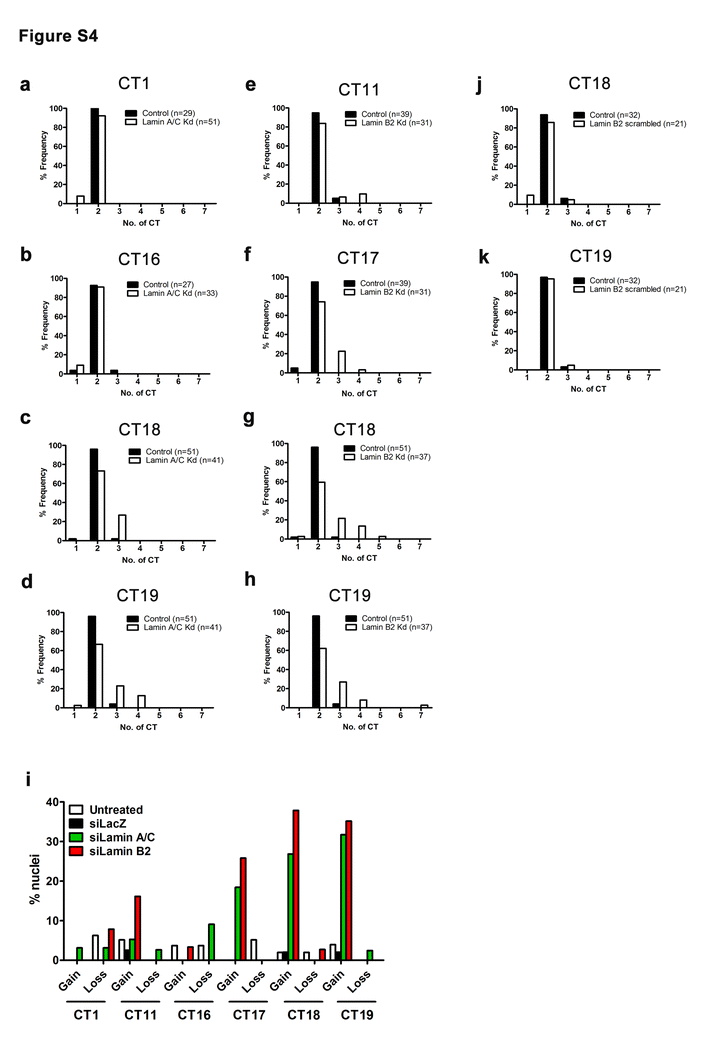

Supplement: Supplementary file 7 — Ploidy of candidate chromosomes upon Lamin depletion in the interphase nucleus. a-d Copy numbers of chromosomes a CT1, b CT16, c CT18 and d CT19 in interphase nuclei upon Lamin A/C depletion in DLD1 cells. Chromosomes 18 and 19 show aneuploidy upon Lamin A/C depletion. e-h Copy numbers of chromosomes e CT11, f CT17, g CT18 and h CT19 in interphase nuclei upon Lamin B2 depletion in DLD1 cells. Chromosomes 11, 17, 18 and 19 are aneuploid upon Lamin B2 depletion. i Plot showing extent of gains and losses of chromosomes 1,11,16,17,18 and 19 upon siLacZ, siLamin A/C and siLamin B2 treatments. ~30-50 nuclei were scored in each set. j-k Copy numbers of chromosomes j CT18 and k CT19 in interphase cells upon transfection with Lamin B2 scrambled oligo, which does not show aneuploidy. (GIF 65 kb) [file 412_2016_580_Fig13_ESM.gif]

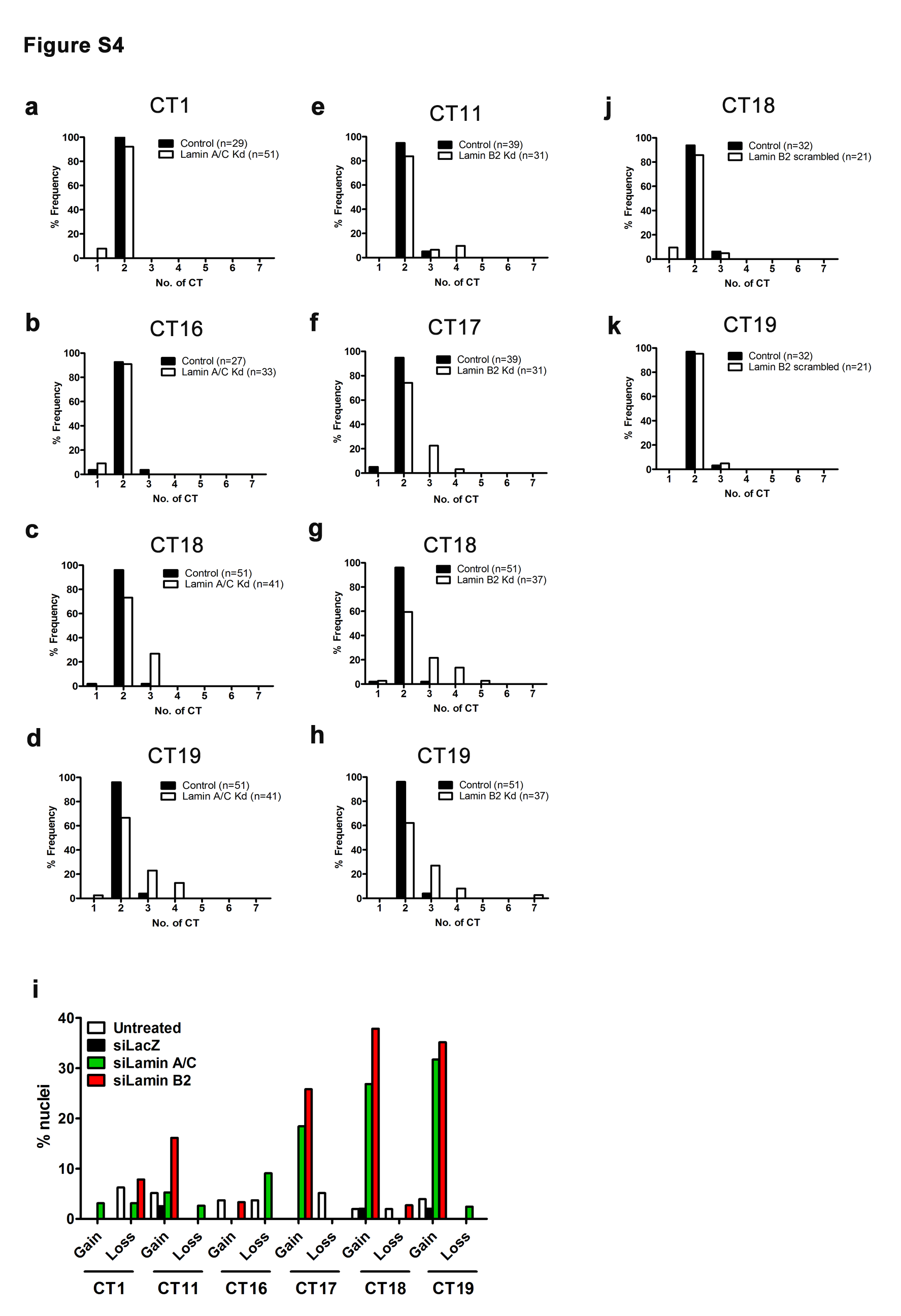

Supplement: Supplementary file 8 — High Resolution Image (TIF 537 kb) [file 412_2016_580_MOESM4_ESM.tif]

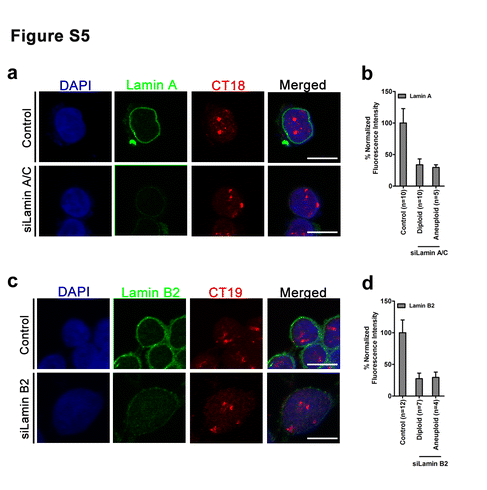

Supplement: Supplementary file 9 — Immuno-FISH for Lamin A-CT18 and Lamin B2-CT19 upon Lamin depletion in DLD1 cells. a Immuno-FISH for Lamin A (green), CT18 (red) in control and Lamin A/C Kd cells. Scale bar ~ 10 μm. b quantification of fluorescence intensities of Lamin A in control and Lamin A/C Kd diploid and aneuploid cells c Immuno-FISH for Lamin B2 (green), CT19 (red) in control and Lamin B2 Kd cells. Scale bar ~ 10 μm. d quantification of fluorescence intensities of Lamin B2 in control and Lamin B2 Kd diploid and aneuploid cells. Comparable level of depletion of Lamin A and B2 was achieved in the diploid and aneuploid nuclei upon Lamin knockdown. (GIF 56 kb) [file 412_2016_580_Fig14_ESM.gif]

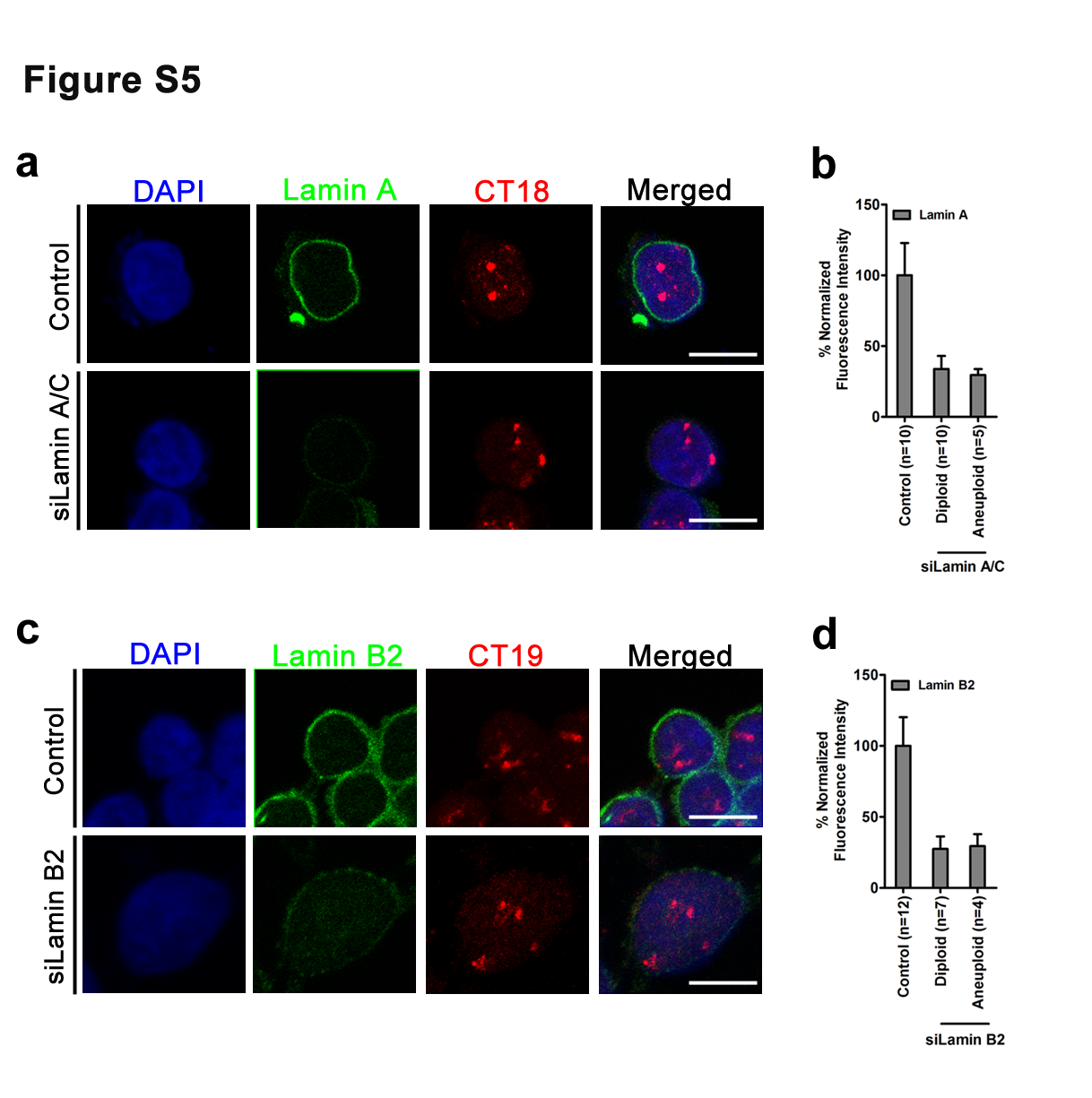

Supplement: Supplementary file 10 — High Resolution Image (TIF 643 kb) [file 412_2016_580_MOESM5_ESM.tif]

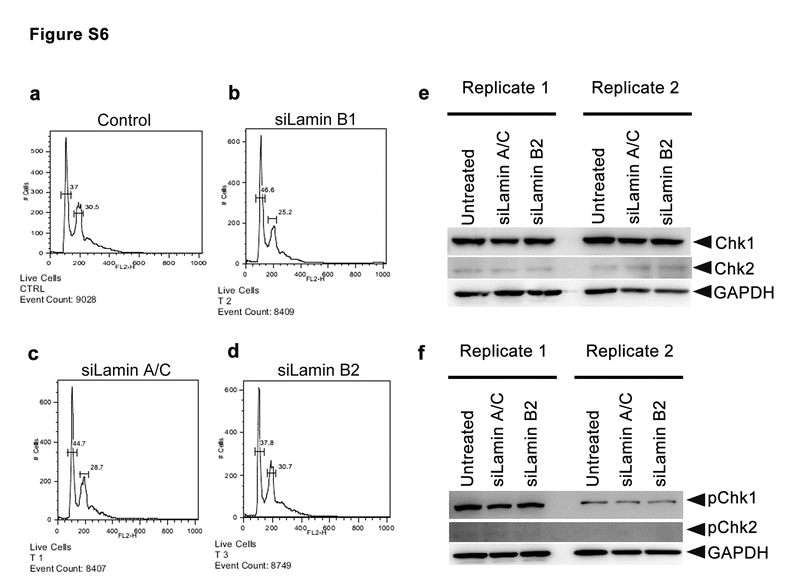

Supplement: Supplementary file 11 — Lamin knockdowns do not alter cell cycle profiles. a-d FACS profiles do not reveal significant changes in overall ploidy levels for a control (untreated), b siLamin B1 c siLamin A/C d siLamin B2 determined using Propidium Iodide staining. e Western Blots showing expression of Chk1 and Chk2 in two biological replicates upon knockdown of Lamin A/C and Lamin B2 in DLD1 cells. No difference in the levels of Chk1 or Chk2 was detected either upon Lamin A/C or B2 depletion. f Western Blots showing expression of pChk1 and pChk2 in two biological replicates upon knockdown of Lamin A/C and Lamin B2 in DLD1 cells. No difference in the levels of pChk1 was detected either upon Lamin A/C or B2 depletion, while pChk2 was hardly detectable. GAPDH was used as a loading control for e and f. (GIF 46 kb) [file 412_2016_580_Fig15_ESM.gif]

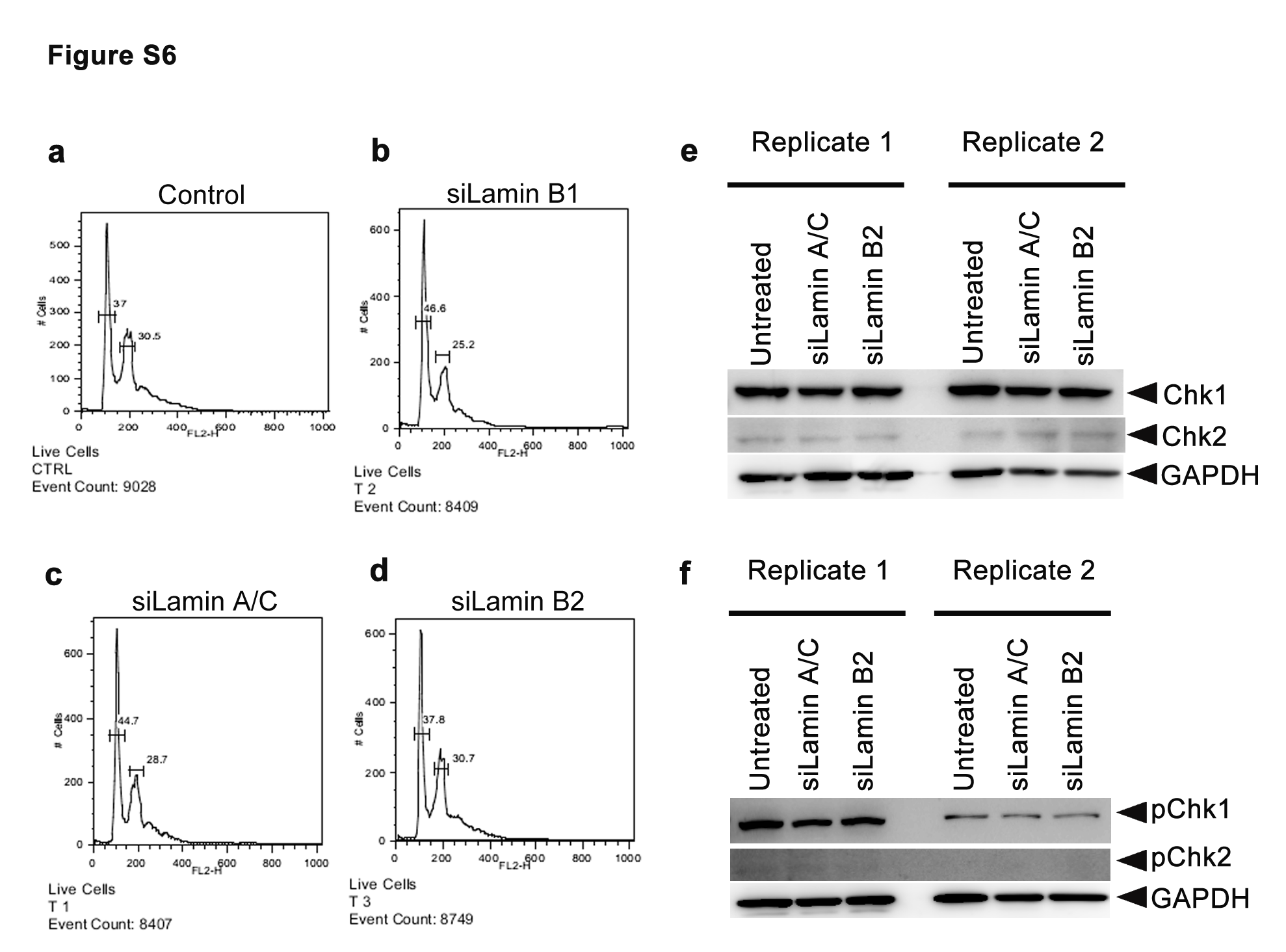

Supplement: Supplementary file 12 — High Resolution Image (TIF 418 kb) [file 412_2016_580_MOESM6_ESM.tif]

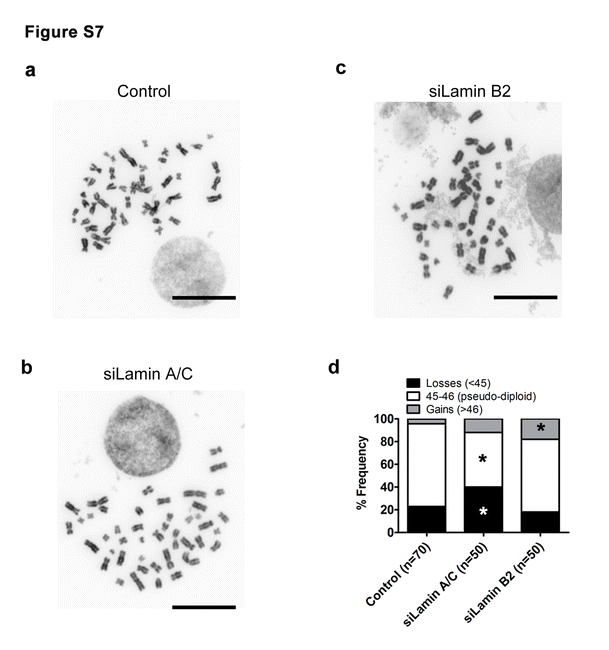

Supplement: Supplementary file 13 — Chromosomal gains and losses upon Lamin A/C, B2 Kd in DLD1 cells. a-c Representative images of inverted DAPI stained metaphase spreads in a control b siLamin A/C c siLamin B2 in DLD1 cells. Scale bar ~ 10 μm. Chromosomes were counted from 50–70 individual metaphase spreads derived from control, Lamin A/C Kd and Lamin B2 Kd cells. d Chromosome losses and gains in DLD1 cells enumerated by counting the number of DAPI stained chromosomes in metaphase spreads from control, Lamin A/C Kd and Lamin B2 Kd. The counts were classified as 45–46 (pseudo-diploid), <45 (losses) and >46 (gains). siLamin A/C: Significant increase in cells with chromosomal losses (p = 0.0345) and significant decrease (p = 0.0047) in the pseudo-diploid population. siLamin B2: significant increase in chromosomal gains (p = 0.018). (GIF 53 kb) [file 412_2016_580_Fig16_ESM.gif]

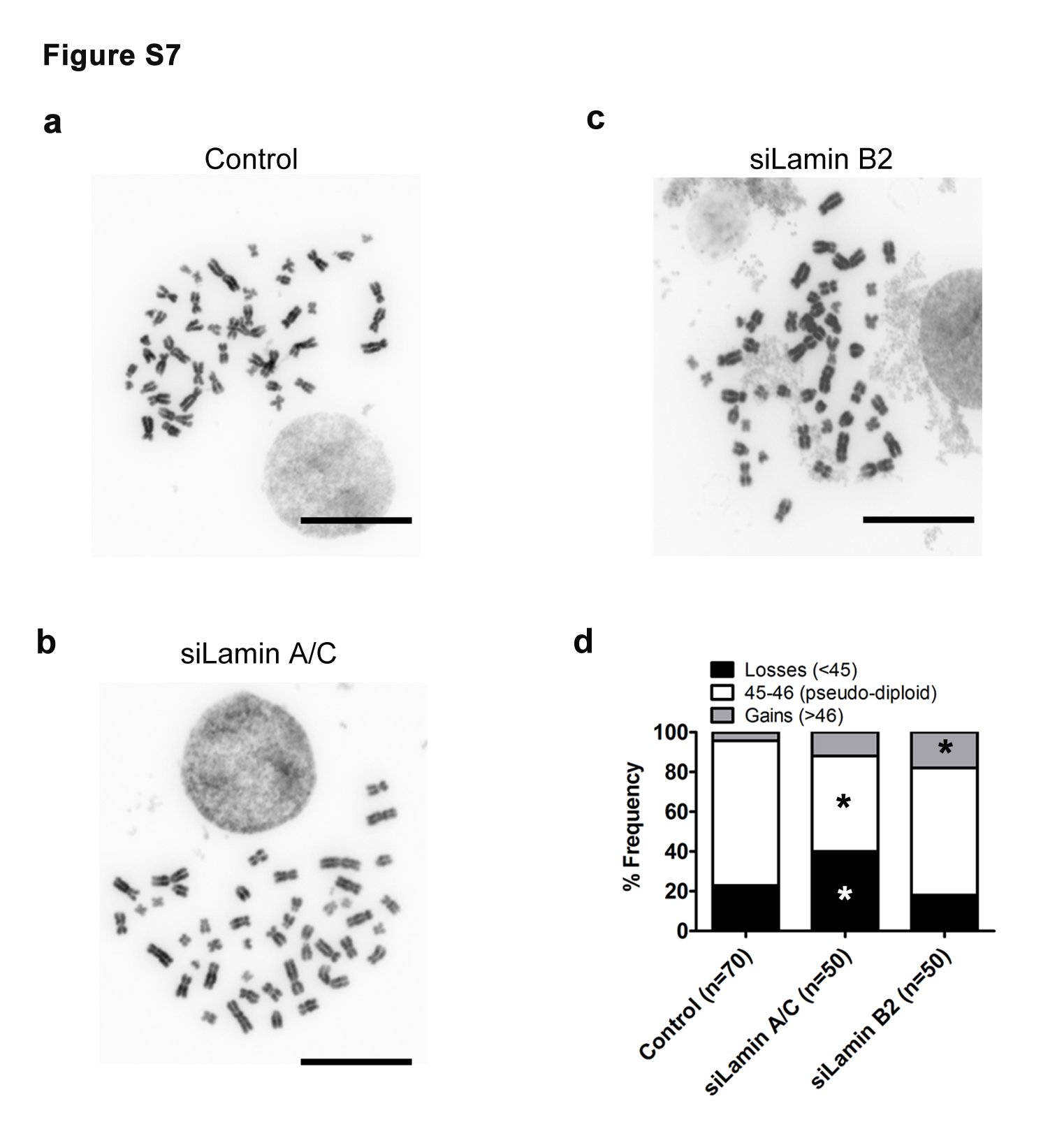

Supplement: Supplementary file 14 — High Resolution Image (TIF 660 kb) [file 412_2016_580_MOESM7_ESM.tif]

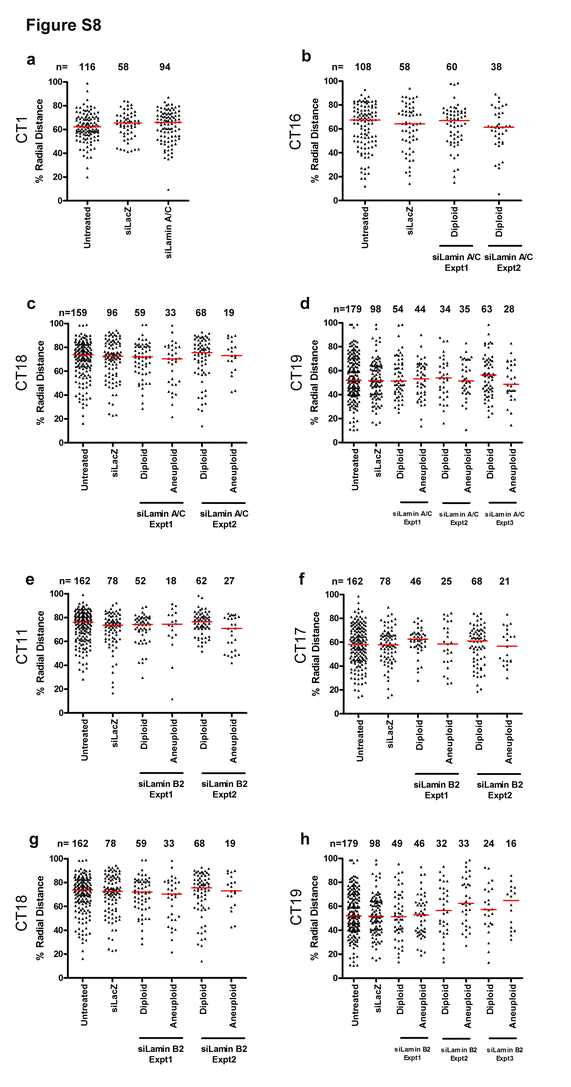

Supplement: Supplementary file 15 — Raw data showing % radial distances of chromosome territories upon Lamin depletion experiment-wise. a-d Each plot represents the radial distance of CT for each independent experiment a CT1 b CT16 c CT18 d CT19 upon siLamin A/C. e-h Each plot represents the radial distance of CT for each independent experiment e CT11 f CT17 g CT18 h CT19 upon siLamin B2. Horizontal bars in the dot scatter plot represent the medians. n = number of chromosome territories quantified. (GIF 91 kb) [file 412_2016_580_Fig17_ESM.gif]

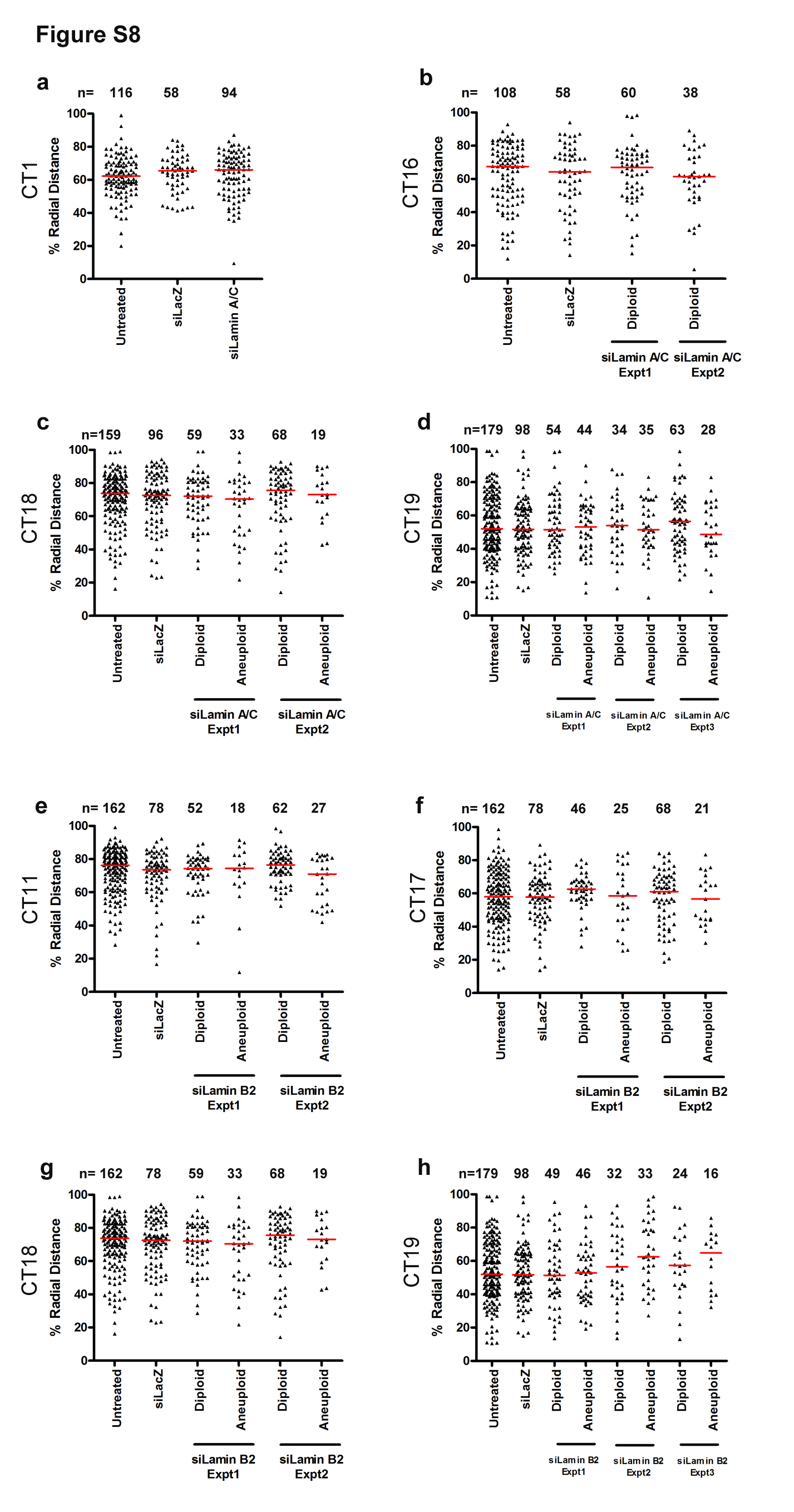

Supplement: Supplementary file 16 — High Resolution Image (TIF 657 kb) [file 412_2016_580_MOESM8_ESM.tif]

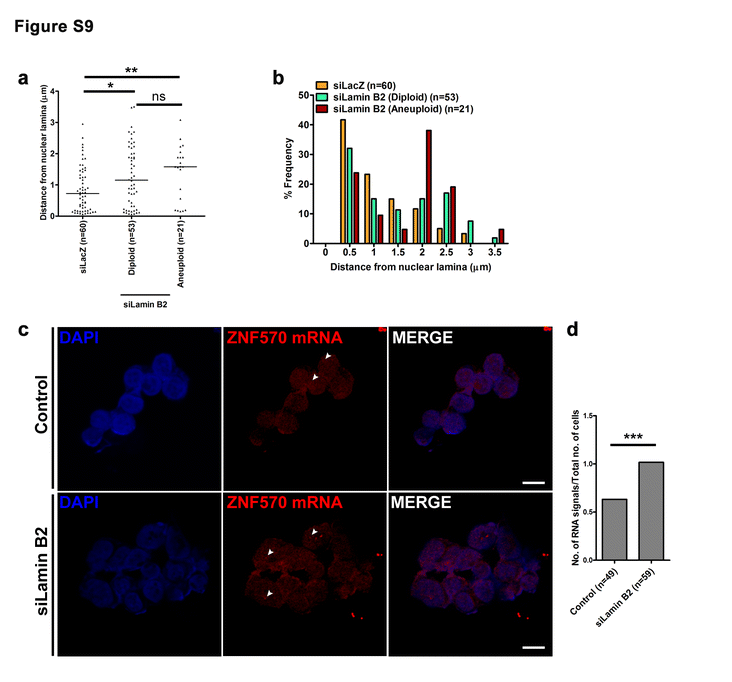

Supplement: Supplementary file 17 — Increase in ZNF570 transcript signals upon Lamin B2 Kd. a Distance of ZNF570 gene locus from the lamina in diploid and aneuploid nuclei upon Lamin B2 Kd. b Distance of ZNF570 from the nuclear lamina plotted in bins of 0.5 μm each from siLacZ, siLamin B2 (diploid and aneuploid cells). Both diploid and aneuploid cells show repositioning of ZNF570 away from the nuclear lamina. c representative RNA-FISH images for ZNF570 (red) in control and siLaminB2 cells. d Quantification of the number of RNA-FISH signals upon total number of cells scored in control and siLamin B2 cells shows an increase in proportion of RNA signals upon siLamin B2. Data compiled from a single experiment. Arrowheads: RNA signals for ZNF570. (GIF 740 kb) [file 412_2016_580_Fig18_ESM.gif]

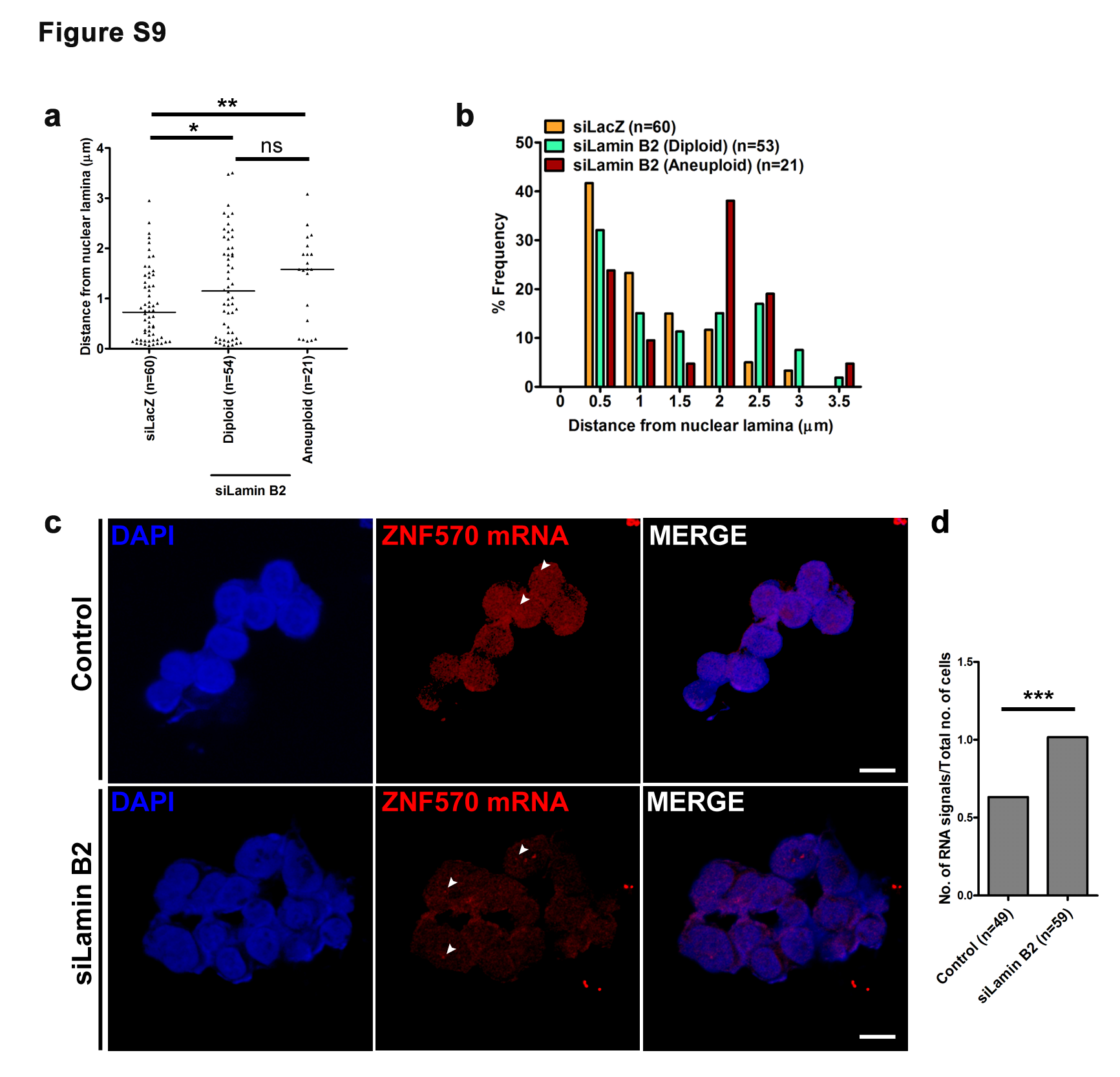

Supplement: Supplementary file 18 — High Resolution Image (TIF 740 kb) [file 412_2016_580_MOESM9_ESM.tif]
