# Supplementary material for: Chromosomal aneuploidies induced upon Lamin B2 depletion are mislocalized in the interphase nucleus
Source: Chromosoma. 2016 Feb 27;126(2):223–44. doi: 10.1007/s00412-016-0580-y (PMC5371638; doi:10.1007/s00412-016-0580-y)
Supplement: Supplementary file 19 — List of qRT-PCR primers used in this study (DOCX 16 kb) [file 412_2016_580_MOESM19_ESM.docx]

**Table S1 – List of all qRT-PCR primers used in this study**

| **Gene Name** | **Primer sequences** | **Efficiency determined at 60ºC** |
| --- | --- | --- |
| LMNA | F- 5’ CCGCAAGACCCTTGACTCA 3’  R- 5’ TGGTATTGCGCGCTTTCAG 3’ | 1.0 |
| LMNB2 | \| F- 5’ AGTTCACGCCCAAGTACATC 3’ \| \| --- \| \| R- 5’ CTTCACAGTCCTCATGGCC 3’ \| | 1.0 |
| ACTIN | F- 5’ GATTCCTATGTGGGCGAC 3’  R-5’ GGTAGTCAGTCAGGTCCCG 3’ | 0.88 |
| AKR1C3-A | F- 5’ GCATGAGGTCTGCCAGAAG 3’  R- 5’ ACCAGCATAGAGCCATCCTC 3’ | 1.00 |
| AKR1C3-B | F- 5’ TGGGAGGCCATGGAGAAG 3’  R- 5’ GAAGTTTGACACCCCAATGGA 3’ | 1.23 |
| MANSC1-A | F- 5’ CCGCAGGAAACGTTACTCA 3’  R – 5’ ACTCATTGCATTTGGGCTTC 3’ | 1 |
| MANSC1-B | F – 5’ TGAAACCAGCAAAAGGACTTATGA 3’  R - 5’ TCTTGGCTTGGCAAATTTCTG 3’ | 1 |
| LRRC47-A | F-5’ GGATGCCCTCATTCTGAAAA 3’  R – 5’ GTGGGATCTGGAAGTTGTCC 3’ | 1.01 |
| LRRC47-B | F- 5’ GGCCTGCACAGATACCTTCAC 3’  R- 5’ CATCCACAAGACACGGGTAATTT 3’ | 1.0 |
| AKR1B10-A | F – 5’ AAACTGGAGGGCCTTTGACT 3’  R – 5’ TTCTGCATCGAAGGGAAAGT 3’ | 1.03 |
| AKR1B10-B | F- 5’ TGGAAGTCTCCTCTTGGCAAA 3’  R – 5’ TGTGCCGATATCCTGCATCA 3’ | 1.11 |
| PTGS2-A | F – 5’ CTATGTGCTAGCCCACAAAGAA 3’  R – 5’ GGTCAGTCTTATGGCACATTCA 3’ | 1.0 |
| PTGS2-B | F- 5’ TGAGTGTGGGATTTGACCAGTATAA 3’  R – 5’ ATTCCGGTGTTGAGCAGTTTTC 3’ | 1.0 |
| HNRPK-A | F – 5’ ATGGTGATCTTGGTGGACCT 3’  R – 3’ TTAATCCGCTGACCACCTTT 3’ | 1.04 |
| HNRPK-B | F – 5’ TGGAAAAGGAGGCAAGAATATTAAG 3’  R – 5’ TGCTGTCTGGGACTGAAACACT 3’ | 1.05 |
| SMTNL2-A | F – 5’ TTCCCTCTGGTGAATTCGAT 3’  R – 5’ ACCTACAATCGCGTGAGGAA 3’ | 0.87 |
| SMTNL2-B | F – 5’ GGCCAGAGTTTGGATCACGAT 3’  R – 5’ GATGCAGGAGTTTGAGGTCTTTC 3’ | 1.0 |
| ABLIM2-A | F- 5’ AAAGTGTTTCAATGTGACAGGA 3’  R – 5’ AACACATCATCATTTCCAAGC 3’ | 1.23 |
| ABLIM2-B | F- 5’ TGTCAGGTGCGGCCAGAT 3’  R – 5’ CGCCGGATGCCAGATG 3’ | 1.26 |
| TMEM204-A | F – 5’ GGATGAGTCTGGGTGACCTC 3’  R – 5’ CCGACATAACAGTAAACGCACA 3’ | 1.39 |
| TMEM204-B | F – 5’ CCATGGCCGCTGCATT 3’  R – 5’ AAAGTCACGAGCCCGATGAC 3’ | 1.23 |
| CDC42-A | F- 5’ ATTTTGGTTGCAGTTTCCAA 3’  R – 5’ TTTGGGAAGGTGGGAAAGAT 3’ | 1.10 |
| CDC42-B | F – 5’ AGAAAAGTGGGTGCCTGAGATAAC 3’  R – 5’ GAGTCCCAACAAGCAAGAAAGG 3’ | 1.08 |
| ITGA2-A | F- 5’ AAGCCGAAGTACCAACAGGA 3’  R – 5’ GCCGAGCTTCCATAAAATTG 3’ | 1.1 |
| ITGA2-B | F- 5’ TGCCCCGAGCACATCAT 3’  R – 5’ ACGCAAATCCAAAGAGTTGACA 3’ | 1.0 |
| TMEM14B-A | F – 5’ AACGTTTGGGGTTTCCTAGC 3’  R – 5’ CAAACTGGCACCTGCAATTA 3’ | 1.391 |
| TMEM14B-B | F- 5’ CTTTGGCTACACAGCACTGGTT 3’  R – 5’ GCACGCTGCCTGTTTTTACA 3’ | 1.23 |
| REG4-A | F – 5’ CCGTCCTCTTCCTTTCTGCT 3’  R – 5’ TGCTAGGTTTCCCCTCTGAA 3’ | 1 |
| REG4-B | F – 5’ GGTTTTACCACAAGTCCAATTGC 3’  R – 5’ ACTCGAGCTCGGCATCAGA 3’ | 1 |
| KPNA7-A | F – 5’ GAAAGAGGCTGTCTGGATGG 3’  R – 5’ AGATTCACCAGTGGCTCCAG 3’ | 1.1 |
| KPNA7-B | F – 5’ CCACCCTGCCGATCACAT 3’  R – 5’ TTCGGCACAGATTCGACAAG 3’ | 1.1 |
| C1orf144-A | F- 5’ GCAATTTTTAGCCAGGGACA 3’  R – 5’ TGGTACATTTCCTGTGTGCAA 3’ | 1.12 |
| C1orf144-B | F- 5’ CACAAAAAGAGAGCAGGAAATCC 3’  R- 5’ GGCTATCGTCCTGAATCACAATG 3’ | 1.2 |
| KRCC1-A | F- 5’ ATCCGTGTTCATACCACTTTT 3’  R – 5’ TCTTTAACTTCTAACAAAGGC 3’ | 1.2 |
| KRCC1-B | F- 5’ CCAGACCCACGTATAGAATGTTTG 3’  R- 5’ TCTTGGGTAGGTCTGGATGGTT 3’ | 1.0 |
| MESDC2-A | F – 5’ GGGGTTCTGTTTTGTTTCCTT 3’  R – 5’ CATATAACCCCTCGACACCAG 3’ | 1.08 |
| MESDC2-B | F – 5’ CAGCCTTTTCAATGCCAACTATG 3’  R – 5’ GCATGAAGATAGCACGGTCTGA 3’ | 1.07 |
| ZNF570 | F - 5' GGCTGGGAGCCTATATGTGA 3’  R- 5' CTTCCTTGAAACACGCCTTC 3' |  |
| GAPDH | F - 5'-CGAGATCCCTCCAAAATCAAG-3'  R - 5'-GCAGAGATGATGACCCTTTTG-3' | 0.81 |
